# Structural insights into ligand recognition and subtype selectivity of the human melanocortin-3 and melanocortin-5 receptors

**DOI:** 10.1038/s41421-023-00586-4

**Published:** 2023-07-31

**Authors:** Wenbo Feng, Qingtong Zhou, Xianyue Chen, Antao Dai, Xiaoqing Cai, Xiao Liu, Fenghui Zhao, Yan Chen, Chenyu Ye, Yingna Xu, Zhaotong Cong, Hao Li, Shi Lin, Dehua Yang, Ming-Wei Wang

**Affiliations:** 1 Department of Pharmacology, School of Basic Medical Sciences, Fudan University, Shanghai, China; 2 Research Center for Deepsea Bioresources, Sanya, Hainan China; 3 State Key Laboratory of Chemical Biology, Shanghai Institute of Materia Medica, Chinese Academy of Sciences, Shanghai, China; 4 The National Center for Drug Screening, Shanghai Institute of Materia Medica, Chinese Academy of Sciences, Shanghai, China; 5 University of Chinese Academy of Sciences, Beijing, China; 6 Department of Chemistry, School of Science, The University of Tokyo, Tokyo, Japan; 7 School of Pharmacy, Hainan Medical University, Haikou, Hainan China

**Keywords:** Cryoelectron microscopy, Cell signalling

## Abstract

Members of the melanocortin receptor (MCR) family that recognize different melanocortin peptides mediate a broad spectrum of cellular processes including energy homeostasis, inflammation and skin pigmentation through five MCR subtypes (MC1R–MC5R). The structural basis of subtype selectivity of the endogenous agonist γ-MSH and non-selectivity of agonist α-MSH remains elusive, as the two agonists are highly similar with a conserved HFRW motif. Here, we report three cryo-electron microscopy structures of MC3R–G_s_ in complex with γ-MSH and MC5R–G_s_ in the presence of α-MSH or a potent synthetic agonist PG-901. The structures reveal that α-MSH and γ-MSH adopt a “U-shape” conformation, penetrate into the wide-open orthosteric pocket and form massive common contacts with MCRs via the HFRW motif. The C-terminus of γ-MSH occupies an MC3R-specific complementary binding groove likely conferring subtype selectivity, whereas that of α-MSH distances itself from the receptor with neglectable contacts. PG-901 achieves the same potency as α-MSH with a shorter length by rebalancing the recognition site and mimicking the intra-peptide salt bridge in α-MSH by cyclization. Solid density confirmed the calcium ion binding in MC3R and MC5R, and the distinct modulation effects of divalent ions were demonstrated. Our results provide insights into ligand recognition and subtype selectivity among MCRs, and expand the knowledge of signal transduction among MCR family members.

## Introduction

The melanocortin system is a key part of the neuro-immune-endocrine axis consisting of five melanocortin receptor (MCR) subtypes (MC1R–MC5R), endogenous agonists (melanocortins) and antagonists (agouti and agouti-related peptide, AgRP)^1^. It mediates a wide and complex array of physiological effects, for example, melanogenesis, immunoregulation, pigmentation, adrenocortical steroidogenesis, energy metabolism, sexual function and exocrine secretion^2–6^. The melanocortins are a family of neuropeptide hormones including three different forms of melanocyte-stimulating hormone (α-MSH, β-MSH and γ-MSH) and adrenocorticotropic hormone (ACTH)^7^. All of them are derived from alternate post-translational modification of *proopiomelanocortin* (*POMC*) gene products and share a conserved tetrapeptide sequence, namely His-Phe-Arg-Trp (HFRW), the key motif for recognition of endogenous ligands by MCRs^8,9^.

The biological actions of melanocortins are executed by MCRs. MC1R is mainly expressed in melanocytes, melanoma cells and immune cells, which controls skin and hair color and modulates immune responses^4,10^. MC2R is mainly located in the adrenal cortex, stimulating the biosynthesis of glucocorticoids^11^. MC4R is known as a neural MCR due to its high expression in various brain regions including hypothalamus, brainstem, and cortex. It engages in energy homeostasis, feeding and sexual behaviors, and male erectile function^12–14^.

In addition to MC4R, MC3R is also widely distributed in the brain, being predominantly expressed in the hypothalamus, mainly the arcuate nucleus and ventromedial hypothalamus, structures that are involved in regulating energy homeostasis, metabolism and appetite^15–17^. Many studies revealed that MC3R and MC4R may function independently, playing complementary rather than redundant roles in the control of energy balance^18,19^.

The last MCR to be cloned and characterized was MC5R, which is expressed extensively in peripheral organs and tissues^20–22^. MC5R appears to play a key role in immune and inflammatory responses, and is essential for temperature control and exocrine function^2,21–23^. Selective MC5R agonists have been shown to exert beneficial effects in immune disorders, metabolic endocrinopathies and other pathological conditions, such as kidney diseases and dry eyes^24^. Selective MC5R antagonists were developed to treat seborrhea and acne vulgaris^25^. Except MC1R, the other four MCRs share 56%–74% sequence similarities but mediate different functions. Cryo-electron microscopy (cryo-EM) structures of MC1R, MC2R and MC4R were reported recently, providing useful insights into the structural basis governing differentiated actions of endogenous and synthetic peptides mediated by these three MCRs^26–30^.

**Fig. 1:**
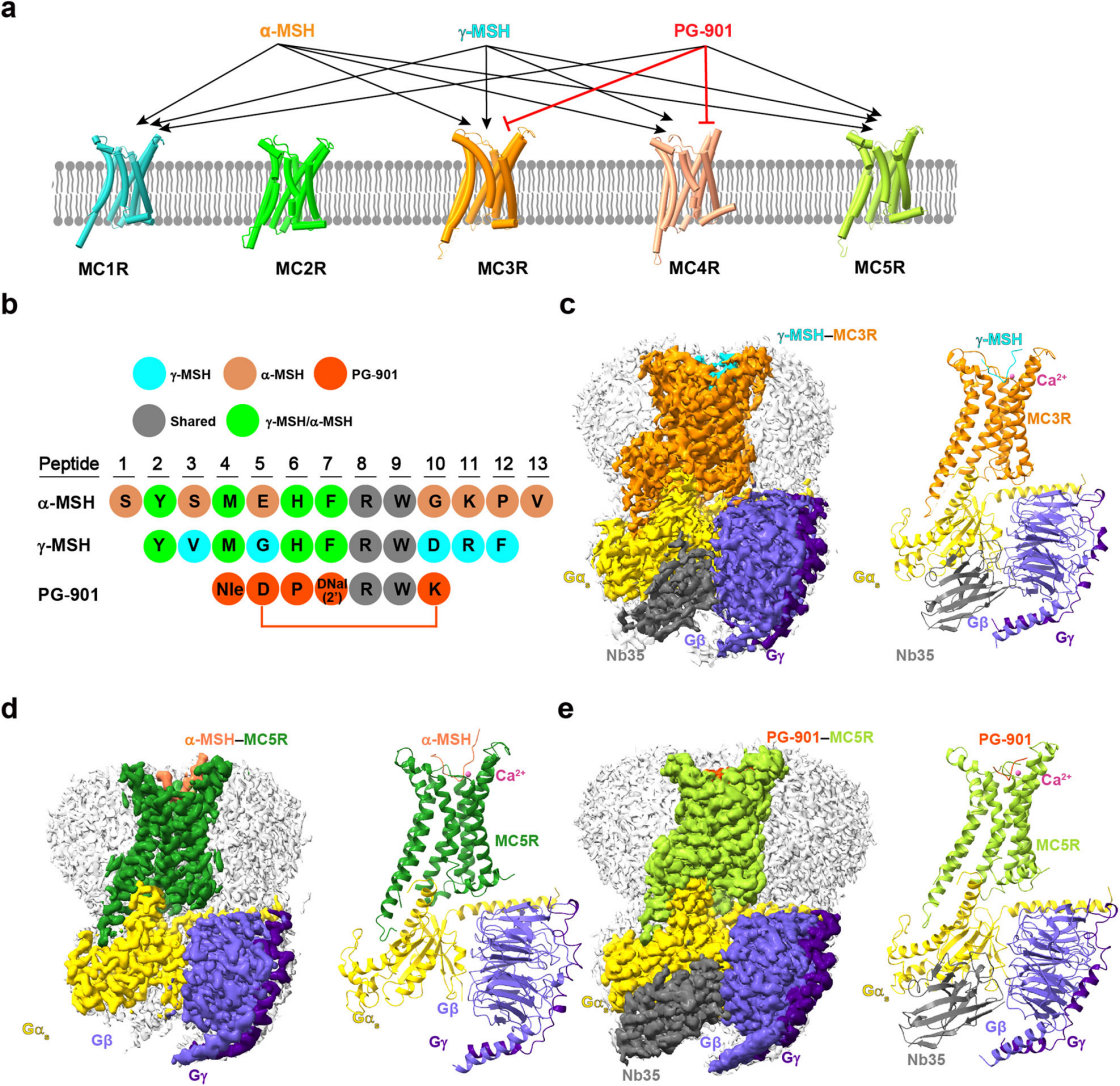
Cryo-EM structures of γ-MSH-bound MC3R, α-MSH- and PG-901-bound MC5R in complex with G_s_. **a** Activities of γ-MSH, α-MSH and PG-901 in the MCRs (MC1R–MC5R). **b** Amino acid sequences of γ-MSH, α-MSH and PG-901. Residues are colored according to sequence conservation among γ-MSH, α-MSH and PG-901. **c**–**e** Cryo-EM density maps (left) and cartoon representation (right) of the γ-MSH–MC3R–G_s_–Nb35 complex (**c**), α-MSH–MC5R–G_s_–Nb35 complex (**d**) and PG-901–MC5R–G_s_–Nb35 complex (**e**). The γ-MSH is shown in cyan, α-MSH in coral and PG-901 in orange red. The corresponding receptors are shown in dark orange, forest green and yellow green, respectively. Ca^2+^ is shown in hot pink, Gα_s_ in gold, Gβ in slate blue, Gγ in indigo and Nb35 in dim gray.

A number of natural and synthetic MCR ligands were characterized or developed. PG-901, a cyclic α-MSH analogue, is the first highly selective agonist of MC5R but also acts as a full antagonist of MC3R^31^ (Fig. 1a, b). Meanwhile, MC3R is the only MCR that could be activated by γ-MSH to the same extent as by any other melanocortins^24,32^. In this study, we determined three single-particle cryo-EM structures of γ-MSH-bound MC3R–G_s_, α-MSH-bound MC5R–G_s_ and PG-901-bound MC5R–G_s_ complexes at global resolutions of 2.86 Å, 2.73 Å and 2.59 Å, respectively. Combined with functional data obtained from site-directed mutagenesis, these structures provide valuable mechanistic information at the near-atomic level concerning ligand recognition and receptor activation for MC3R and MC5R, useful to better understand the subtype selectivity and rational design of novel therapeutic agents targeting MCRs.

## Results

### Overall structures of MC3R–G_s_ and MC5R–G_s_ complexes

After sample preparation, cryo-EM data collection and analysis, three-dimensional (3D) consensus density maps were reconstructed with overall resolutions of 2.86 Å, 2.73 Å and 2.59 Å for the γ-MSH–MC3R–G_s_, α-MSH–MC5R–G_s_ and PG-901–MC5R–G_s_ complexes, respectively (Fig. 1; Supplementary Figs. S1, S2 and Table S1). The cryo-EM maps allowed us to build near-atomic models for most regions of the complexes except for the flexible α-helical domain (AHD) of Gα_s_, the N-termini of the receptors (M1 to F34 in MC3R and M1 to G37 in MC5R) and the intracellular loop 3 (ICL3) (A227 to Q233 in MC3R and S227 to Q230 in MC5R) (Supplementary Fig. S3). Because of the relatively high resolution of the three structures, α-MSH, PG-901 and γ-MSH were well-defined in the EM density maps. Besides, the calcium ion (Ca^2+^) densities were also clearly seen in the three complex maps (Fig. 1c–e).

**Fig. 2:**
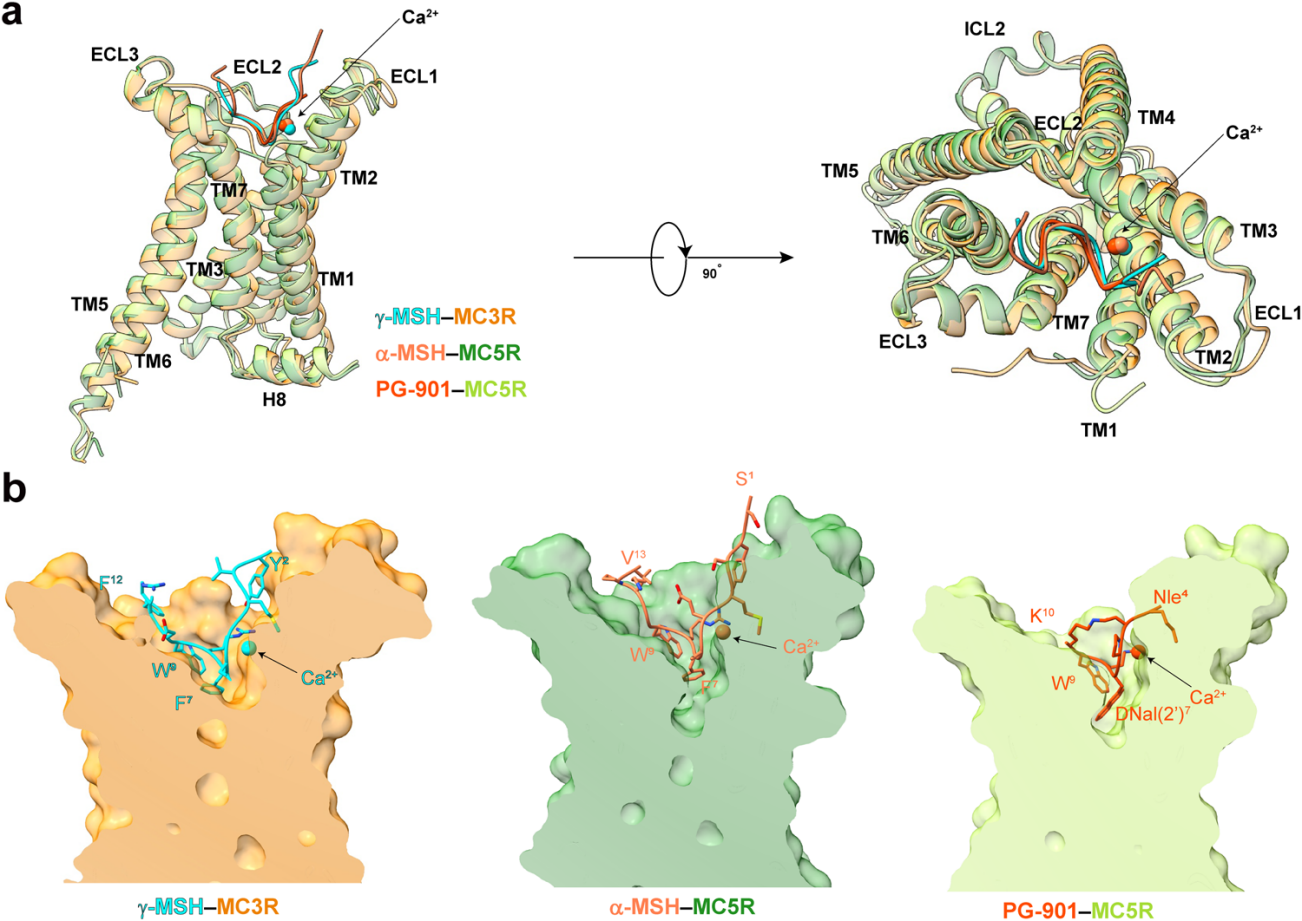
Structural comparison of active MC3R and MC5R. **a** Superimposition of the γ-MSH–MC3R, α-MSH–MC5R and PG-901–MC5R complexes. G protein is omitted for clarity. **b** Surface representation of ligand-binding pockets among γ-MSH-bound MC3R (left), α-MSH-bound MC5R (middle) and PG-901-bound MC5R (right). Ca^2+^ is shown as sphere.

As shown in Fig. 2a, the cryo-EM structures of α-MSH–MC5R–G_s_ and PG-901–MC5R–G_s_ closely resembled that of γ-MSH–MC3R–G_s_ complex with Cα root mean square deviation (RMSD) values of 0.57 Å and 0.68 Å, respectively. When comparing the overall structures across the MCR subfamily, we found that these three structures displayed high degrees of resemblance relative to previously reported MC1R/MC2R/MC4R structures with Cα RMSD values ranging from 0.54 Å to 1.04 Å (PDB codes: 7F4D (α-MSH–MC1R–G_s_), 8GY7 (ACTH–MC2R–G_s_–MRAP1) and 7F53 (α-MSH–MC4R–G_s_))^26–28^. The bound α-MSH, PG-901 and γ-MSH all adopted a “U-shape” conformation in the ligand-binding pocket, while the conserved motif X–X–R–W deeply inserted into the transmembrane domain (TMD) core. Of them, the aromatic moieties of F^7^ (superscript indicates the peptide position number based on α-MSH) in γ-MSH, F^7^ in α-MSH and 3-(2-Naphthyl)-d-alanine (DNal(2’))^7^ in PG-901 inserted deepest within the receptor core (Fig. 2b).

Unlike other class A G protein-coupled receptors (GPCRs), MC3R and MC5R have extremely short (three amino acids) extracellular loop 2 (ECL2) lacking the two class A GPCR-conserved cysteines at 3.25 (Ballesteros–Weinstein numbering)^33^ and 45.50 (the most conserved residue in ECL2) that lock the extracellular tip of transmembrane helix (TM) 3 and ECL2^34^. Instead, solid cryo-EM density map supports an MCR-conserved intra-ECL3 disulfide bond for MC3R (C268^ECL3^–C274^ECL3^) and MC5R (C264^ECL3^–C270^ECL3^), thereby allowing the formation of an α-helix for most of ECL3 (Fig. 2a). Besides, another disulfide bond was only found in MC3R (between C35 in the N-terminus and C276 in ECL3) but not in MC5R whose equivalent residues are C33 and R272. Such an orientation of the receptor extracellular face allows the bound peptide to form massive interactions with the MCRs (Supplementary Tables S2–S4), as indicated by the large interface areas (1869 Å^2^ for γ-MSH–MC3R and 1930 Å^2^ for α-MSH–MC5R). Alternatively, PG-901, which is composed of only seven amino acids but displays a similar potency as α-MSH (Fig. 1b; Supplementary Fig. S1h, k), has fewer contacts with MC5R with an interface area of 894 Å^2^.

### Molecular recognition of γ-MSH

**Fig. 3:**
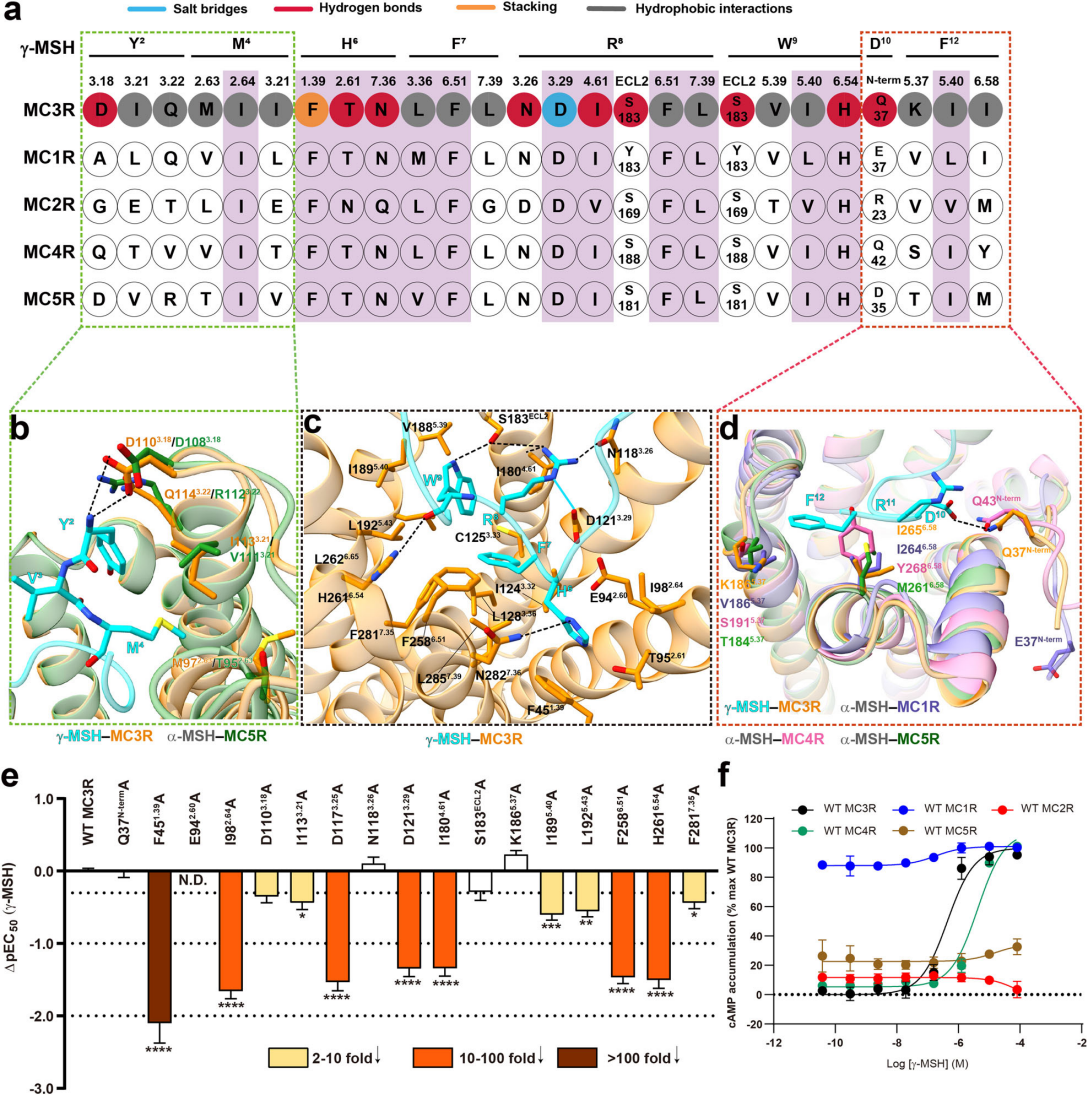
Molecular recognition of γ-MSH. **a** Schematic diagram of interactions between γ-MSH and MC3R. Amino acid residues of MC3R are colored blue for salt bridge, red for hydrogen bond, yellow for stacking and gray for hydrophobic interactions. Similar residues around the binding pocket in five MCRs are highlighted in pink. **b** Interactions of the N-terminus of γ-MSH (cyan) with MC3R TMD (dark orange). The aligned receptor residues in MC5R TMD (α-MSH is omitted for clarity) are shown for comparison. The hydrogen bonds are shown as black dashed lines. **c** Interactions of the middle region of γ-MSH with MC3R. The hydrogen bonds and salt bridges are shown as black dashed lines and cyan solid lines, respectively. **d** Interactions of the C-terminus of γ-MSH with MC3R. The receptor residues in MC1R (slate blue), MC4R (hot pink) and MC5R (forest green) are shown for comparison. The α-MSH is omitted for clarity. **e** Effects of MC3R mutations on γ-MSH-induced cAMP accumulation. Bars represent differences in the calculated γ-MSH potency (pEC_50_) for representative mutants relative to the wild type (WT). Data are colored according to the extent of effect. Data shown are from at least three independent experiments performed in quadruplicate. All data were analyzed by one-way ANOVA and Dunnett’s test. *P < 0.05, **P < 0.01, ***P < 0.001, and ****P < 0.0001. N.D. not determined. **f** Subtype selectivity of γ-MSH at MCRs. Data are shown as means ± SEM of at least three independent experiments performed in quadruplicate.

γ-MSH is an endogenous agonist of four MCRs except MC2R and preferentially activates MC3R and MC1R with superior potencies over other MCRs including MC5R in our functional assay (Fig. 3). According to the peptide sequence conservation, γ-MSH could be divided into three segments: the conserved HFRW motif in the middle, four less conserved residues (Y^2^‒G^5^) at the N-terminus and three non-conserved residues (D^10^‒F^12^) at the C-terminus (Fig. 1b). These three segments have distinct roles in ligand recognition correlated with subtype selectivity (Fig. 3).

The residues that interacted with the central HFRW motif are highly conserved among MCRs (Fig. 3a). Specifically, H^6^ of γ-MSH was stacked by a conserved aromatic residue F45^1.39^ in MC3R, and the substitution of F45^1.39^ to alanine reduced the potency and binding affinity of γ-MSH by 126-fold and 48-fold, respectively (Fig. 3c; Supplementary Tables S5, S6). F^7^ deeply inserted into a hydrophobic cavity consisting of L128^3.36^, F258^6.51^ and L285^7.39^, while R^8^ contributed multiple polar contacts including a salt bridge with D121^3.29^ and two hydrogen bonds with N118^3.26^ and I180^4.61^. Another two hydrogen bonds were found between W^9^ and two conserved residues S183^ECL2^ and H261^6.54^ (Fig. 3c). These observations received the support of our mutagenesis studies, where mutants D121^3.29^A, I180^4.61^A, F258^6.51^A and H261^6.54^A decreased the potency of γ-MSH in inducing cAMP signaling by 22-fold, 22-fold, 30-fold and 32-fold, respectively (Fig. 3c; Supplementary Tables S5, S6).

As for the N-terminus of γ-MSH, Y^2^ formed a strong polar interaction with the side chain of D110^3.18^ in MC3R, and M^4^ pointed towards the TM2–TM3 cleft with the formation of hydrophobic contacts with M97^2.63^, I98^2.64^ and I113^3.21^. Consistently, alanine replacement of I98^2.64^ severely decreased the potency and affinity of γ-MSH (Fig. 3e; Supplementary Tables S5, S6). Notable conformational difference was observed at the C-terminus of the peptide and its interacting receptor extracellular surface. Because the MC1R/MC2R/MC3R-specific disulfide bond (C35‒C276^ECL3^) that connects receptor N-terminus and ECL3 was absent in either MC4R or MC5R, the MC3R N-terminus stabilizes γ-MSH binding by one hydrogen bond (Q37‒D^10^). As a comparison, the N-termini of α-MSH-bound and PG-901-bound MC5R were unstructured. The C-terminal residue F^12^ of γ-MSH was in close contact with MC3R TMs 5/6 residues including K186^5.37^, I189^5.40^ and I265^6.58^. As indicated by MCR sequence alignment (Fig. 3a), the equivalent residues of MC3R I265^6.58^ at MC2R (M242^6.58^), MC4R (Y268^6.58^) and MC5R (M261^6.58^) have significantly larger side chains and may adversely affect γ-MSH binding (Fig. 3d). These observations receive support of our mutagenesis studies where the MC3R mutants I265^6.58^M, I265^6.58^Y and I265^6.58^W decreased the potency of γ-MSH by 3-fold, 4-fold and 18-fold, respectively (Supplementary Fig. S4). Consistently, Y268^6.58^I mutation in MC4R increased the affinity of [Nle^4^] Lys-γ-MSH binding to MC4R^35^.

### Molecular recognition of α-MSH

**Fig. 4:**
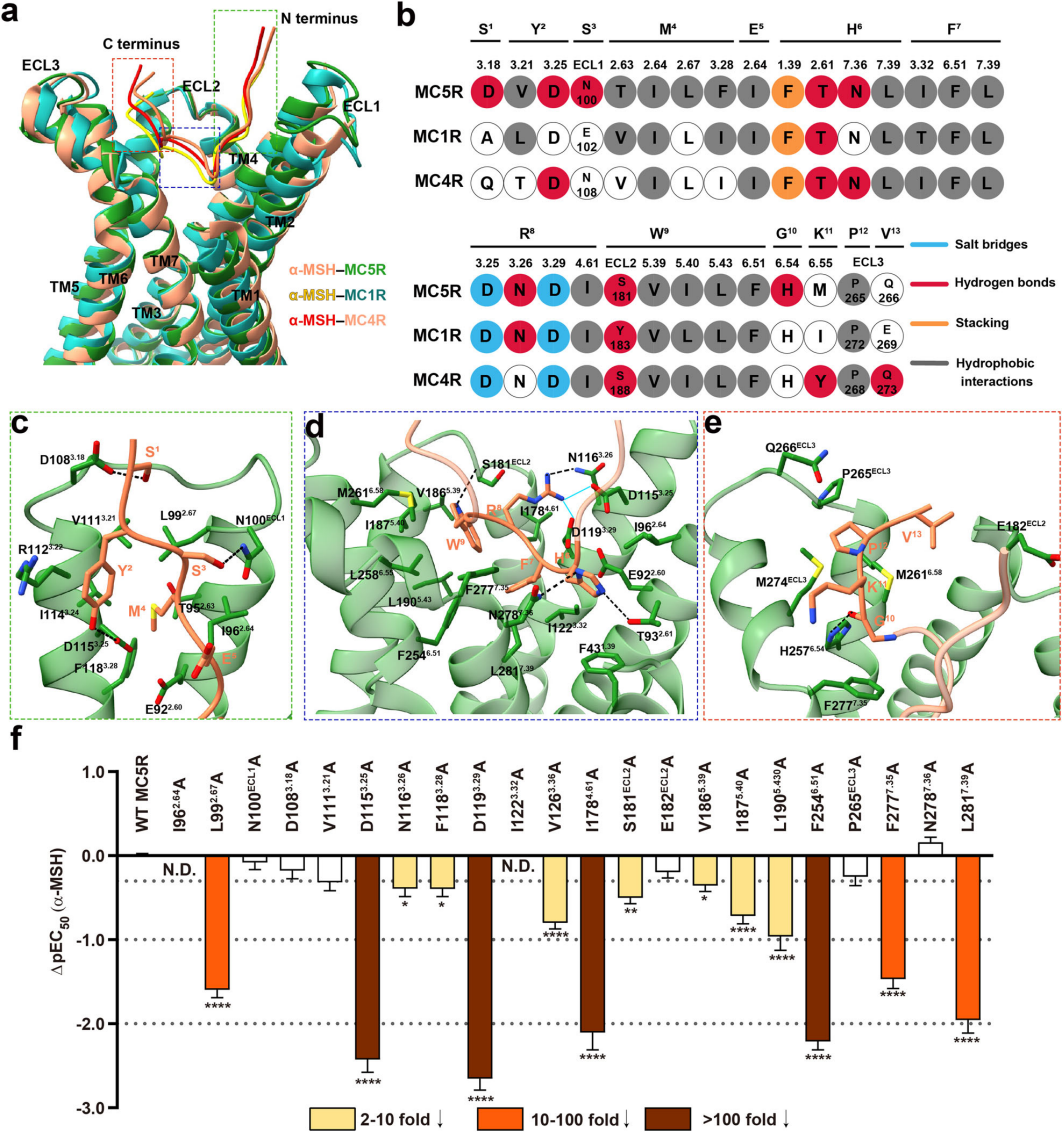
Molecular recognition of α-MSH by MC5R. **a** Structural comparison of G_s_-coupled α-MSH–MC5R, α-MSH–MC1R (PDB code: 7F4D) and α-MSH–MC4R (PDB code: 7F53). The N-termini of MC1R and MC4R and G proteins are omitted for clarity. **b** The peptide recognition modes are described by fingerprint strings encoding different interaction types of the surrounding residues in each receptor. Residues that show no interaction with peptides are displayed as white circles. **c**–**e** Interactions of the N-terminus (**c**), middle region (**d**) and C-terminus (**e**) of α-MSH (coral) with MC5R TMD (forest green). Residues involved in the interactions are shown as sticks. The hydrogen bonds and salt bridges are shown as black dashed lines and cyan solid lines, respectively. **f** Effects of MC5R mutations on α-MSH-induced cAMP accumulation. Bars represent differences in the calculated α-MSH potency (pEC_50_) for representative mutants relative to the wild type (WT). Data are colored according to the extent of effect. Data shown are from at least three independent experiments performed in quadruplicate. All data were analyzed by one-way ANOVA and Dunnett’s test. *P < 0.05, **P < 0.01, ***P < 0.001, and ****P < 0.0001. N.D. not determined.

Unlike γ-MSH, α-MSH is a non-selective full agonist of MC1R, MC3R, MC4R and MC5R but not MC2R. Superimposition of the TMDs of α-MSH-bound MC5R, MC1R^27^ and MC4R^26^ showed that the α-MSH molecules were well-overlapped especially the middle region (H^6^‒F^7^‒R^8^‒W^9^) and recognized by MCR-conserved residues in the lower half of the TMD pocket (Fig. 4a, b). Meanwhile, several MC5R-specific movements were observed. Relative to MC1R, the inward movement of the extracellular tip of MC5R TM2 (by 2.8 Å as measured by Cα atoms of L99^2.67^ (MC5R) and L101^2.67^ (MC1R)) shifted the N-terminus of α-MSH inward by 1.3 Å (measured by Cα of Y^2^). The ECL3 of MC5R moved outward by 1.8 Å relative to MC1R (measured by Cα of P265 in MC5R and Cα of P268 in MC1R), resulting in a shift of the C-terminus of α-MSH by 2.2 Å towards ECL3 (measured by Cα of V^13^) (Fig. 4a).

The α-MSH binding was stabilized by a series of polar and nonpolar interactions involving all TMs and ECLs of MC5R (Fig. 4a–e; Supplementary Table S3). The N-terminus of α-MSH adopted a vertical conformation along with TM2 and TM3, forming two hydrogen bonds (S^1^‒D108^3.18^ and S^3^‒N100^ECL1^) (Fig. 4c). For the middle region, H^6^ packed with F43^1.39^; F^7^ was surrounded by hydrophobic residues; R^8^ formed polar contacts with D115^3.25^, N116^3.26^ and D119^3.29^; and W^9^ was fully buried by the TM5/TM6 residues (Fig. 4d). These observations were in line with the mutagenesis studies, where I122^3.32^A mutation in MC5R completely abolished α-MSH-induced cAMP response, while D115^3.25^A, D119^3.29^A and F254^6.54^A greatly impaired the α-MSH potency (> 100-fold) (Fig. 4f; Supplementary Table S7). The C-terminus of α-MSH was mainly clasped by ECL3, TM6 and TM7 of MC5R with an interface area of 317 Å^2^, significantly smaller than that between the C-terminus of γ-MSH and MC3R (409 Å^2^) (Fig. 4e; Supplementary Table S3).

### Molecular recognition of PG-901

**Fig. 5:**
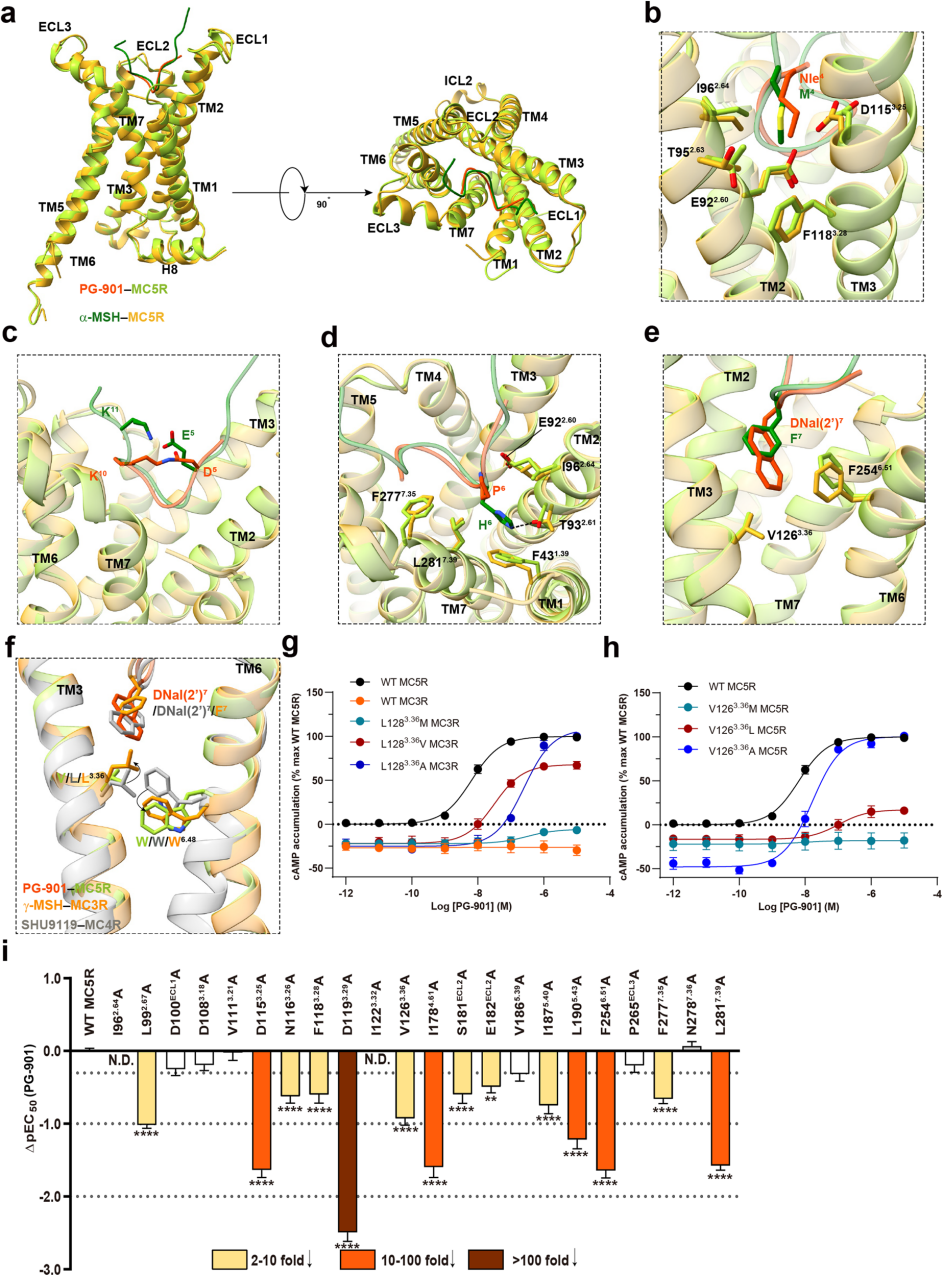
Binding mode comparison between PG-901 and α-MSH at MC5R. **a** Structural comparison of PG-901–MC5R and α-MSH–MC5R. G protein is omitted for clarify. **b**–**e** Comparison of the interactions between Nle^4^ (**b**), cyclized D^5^–K^10^ (**c**), P^6^ (**d**) and DNal(2’)^7^ (**e**) of PG-901 and corresponding residues of α-MSH with MC5R. Residues involved in interactions are shown as sticks. **f** Structural comparison of PG-901–MC5R, γ-MSH–MC3R and SHU9119–MC4R (PDB code: 6W25). **g** Effects of MC3R mutations on PG-901-induced cAMP accumulation. **h** Effects of MC5R mutations on PG-901-induced cAMP accumulation. **i** Effects of MC5R mutations on PG-901-induced cAMP accumulation. Bars represent differences in calculated PG-901 potency (pEC_50_) for representative mutants relative to the wild type (WT). Data are colored according to the extent of effect. Data shown are from at least three independent experiments performed in quadruplicate. All data were analyzed by one-way ANOVA and Dunnett’s test. *P < 0.05, **P < 0.01, ***P < 0.001, and ****P < 0.0001. N.D. not determined.

PG-901 is a synthetic analogue of α-MSH, where the conserved HFRW motif was replaced by P–DNal(2’)–R–W (Fig. 1b). Although the overall structures of PG-901–MC5R–G_s_ and α-MSH–MC5R–G_s_ complexes are highly similar, several PG-901-specific structural features were observed (Fig. 5; Supplementary Table S4). The position of Nle^4^ of PG-901 overlapped well with the M^4^ in α-MSH, while the former with a more hydrophobic side chain received stronger hydrophobic contacts from E92^2.60^, T95^2.63^, I96^2.64^, L99^2.67^, V111^3.21^, D115^3.25^ and F118^3.28^ (Fig. 5b; Supplementary Table S4). L99^2.67^A, D115^3.25^A and F118^3.28^A mutations reduced the PG-901-induced cAMP accumulation by 10-fold, 43-fold and 4-fold, respectively (Fig. 5i; Supplementary Tables S7, S8). The cyclization residues (D^5^–K^10^) aligned well with the E^5^ and K^11^ of α-MSH which formed an intracellular salt bridge and had neglectable contacts with receptor residues (Fig. 5c). The third residue P^6^ in PG-901 failed to form any polar or nonpolar interaction with the receptor; as a comparison, H^6^ in α-MSH made interactions with F43^1.39^, T93^2.61^ and N278^7.36^ (Fig. 5d). However, the side chain of DNal(2’)^7^ in PG-901 is significantly larger than that of α-MSH (F^7^) and deeply inserted into the binding pocket core forming hydrophobic contacts with V126^3.36^ and stacking with F254^6.51^. This observation is absent in the α-MSH–MC5R complex (Fig. 5e, f). Consistently, V126^3.36^A and F254^6.51^A decreased the potency of PG-901 by 8-fold and 44-fold, respectively (Fig. 5e, i; Supplementary Tables S4, S7 and S8). Relevant mutagenesis studies found that MC3R mutants L128^3.36^V and L128^3.36^A elicited significantly better cAMP responses (EC_50_ = 0.032 μM and 0.25 μM, respectively) than the wild type (WT), thereby converting PG-901 from an antagonist to an agonist (Fig. 5g). As a comparison, the PG-901-induced cAMP responses were completely abolished in MC5R mutant V126M and significantly reduced in mutant V126L (by 16-fold; Fig. 5h). These results demonstrate the importance of the DNal(2’)^7^–V126^3.36^ interaction in receptor activation.

### Distinct effects of divalent ions

**Fig. 6:**
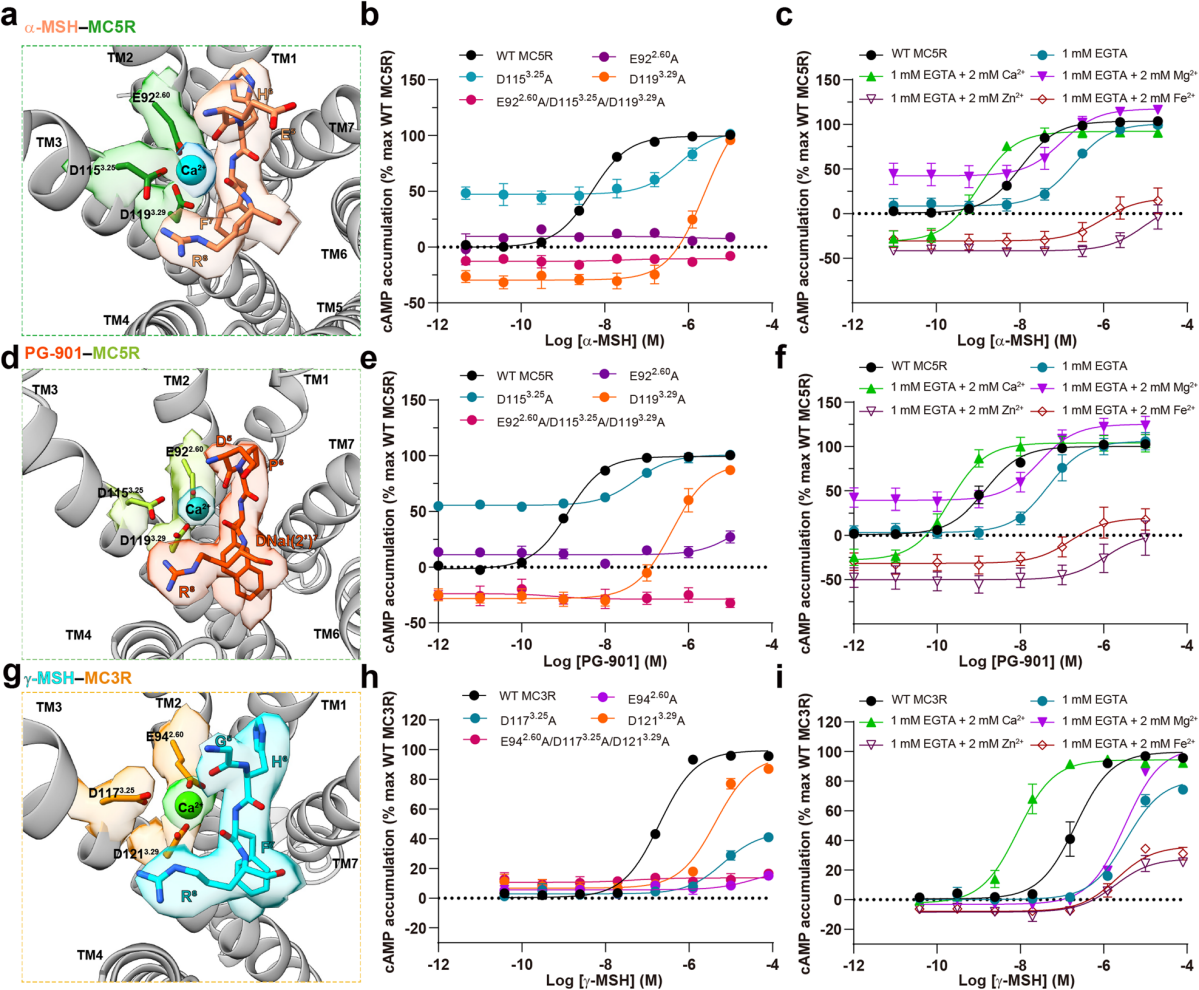
Ca^2+^ binding in MC3R and MC5R. **a** Close-up view of the Ca^2+^-binding pocket in α-MSH-bound MC5R complex. **b**, **c** Effects of MC5R mutations (**b**) and different divalent ions (**c**) on α-MSH-induced cAMP accumulation. **d** Close-up view of Ca^2+^-binding pocket in PG-901-bound MC5R complex. **e**, **f** Effects of MC5R mutations (**e**) and different divalent ions (**f**) on PG-901-induced cAMP accumulation. **g** Close-up view of Ca^2+^-binding pocket in γ-MSH-bound MC3R complex. **h**, **i** Effects of MC3R mutations (**h**) and different divalent ions (**i**) on γ-MSH-induced cAMP accumulation. Data shown are from at least three independent experiments performed in quadruplicate. The cryo-EM density maps of Ca^2+^ and its coordinating residues are shown as colored surface at 0.1 threshold.

Divalent ions have been shown to play an important role in regulating ligand affinities and potencies in MCRs^27,28,36–38^. Here, Ca^2+^ and its coordinating residues were clear enough in the cryo-EM maps for structural modeling, thereby demonstrating that Ca^2+^ resides centrally in a pocket formed by TM2, TM3 and the peptidic agonists in the three structures (Fig. 6). However, the possibility of the presence of other ions at the Ca^2+^ site cannot be ruled out due to the limitation of map resolution and experimental conditions. Specifically, the Ca^2+^ was closely clasped by three negatively charged residues (E^2.60^, D^3.25^ and D^3.29^) in MC3R and MC5R, as well as the peptide residues (G^5^/F^7^/R^8^ in γ-MSH, E^5^/F^7^/R^8^ in α-MSH and D^5^/DNal(2’)^7^/R^8^ in PG-901) (Fig. 6a, d, g). This observation was in line with the abolishment of ligand-binding affinity and potency caused by a triple mutant (E^2.60^A/D^3.25^A/D^3.29^A) (Fig. 6b, e, h; Supplementary Tables S5–S8). For single-point mutation, E92^2.60^A and D119^3.29^A in MC5R completely abolished or greatly reduced (by 447-fold) α-MSH-induced cAMP accumulation, whereas D115^3.25^A increased the basal activity but decreased the α-MSH potency by 269-fold (Fig. 6b). Similar phenomena were also observed for PG-901, indicating a conserved role of Ca^2+^ in MC5R-mediated signal transduction (Fig. 6e). The equivalent mutations in MC3R abolished (E94^2.60^A) or decreased the γ-MSH potency by 34-fold (D117^3.25^A) and 22-fold (D121^3.29^A), without altering the basal activity (Fig. 6h). Replacement of the aspartate/glutamate at these sites by asparagine/glutamine (E^2.60^Q, D^3.25^N and D^3.29^N) in MC3R and MC5R led to similar reduction in cAMP accumulation, indicative of an important role of the charge–charge interaction in maintaining Ca^2+^ binding (Supplementary Fig. S5a–e).

The distinct effects of divalent ions (Ca^2+^, zinc ion (Zn^2+^), magnesium ion (Mg^2+^) and ferrous ion (Fe^2+^)) on ligand-induced cAMP accumulation were investigated for MC3R and MC5R in a divalent ion-free background (Fig. 6c, f, i; Supplementary Fig. S5f, g). In the presence of 1 mM Ca^2+^, the potencies of α-MSH/PG-901 at MC5R, γ-MSH at MC3R/MC1R, and ACTH at MC2R were increased by 182-fold, 230-fold, 407-fold, 294-fold and 6309-fold, respectively, indicating distinct participation of Ca^2+^ in the ligand recognition (Fig. 6c, f, i; Supplementary Fig. S5f, g). Different from acting as a positive allosteric modulator of MC1R^36^, Zn^2+^ addition (1 mM) caused a dramatic reduction of cAMP response as observed in both MC3R and MC5R (Fig. 6c, f, i). Similar reduction was also observed for Fe^2+^ which showed moderate effects (by 0.6-fold for γ-MSH at MC3R, 5-fold for α-MSH at MC5R, and 4-fold for PG-901 at MC5R). The addition of 1 mM Mg^2+^ had neglectable impact on the G_s_ signaling pathway.

### G protein coupling of MC3R and MC5R

**Fig. 7:**
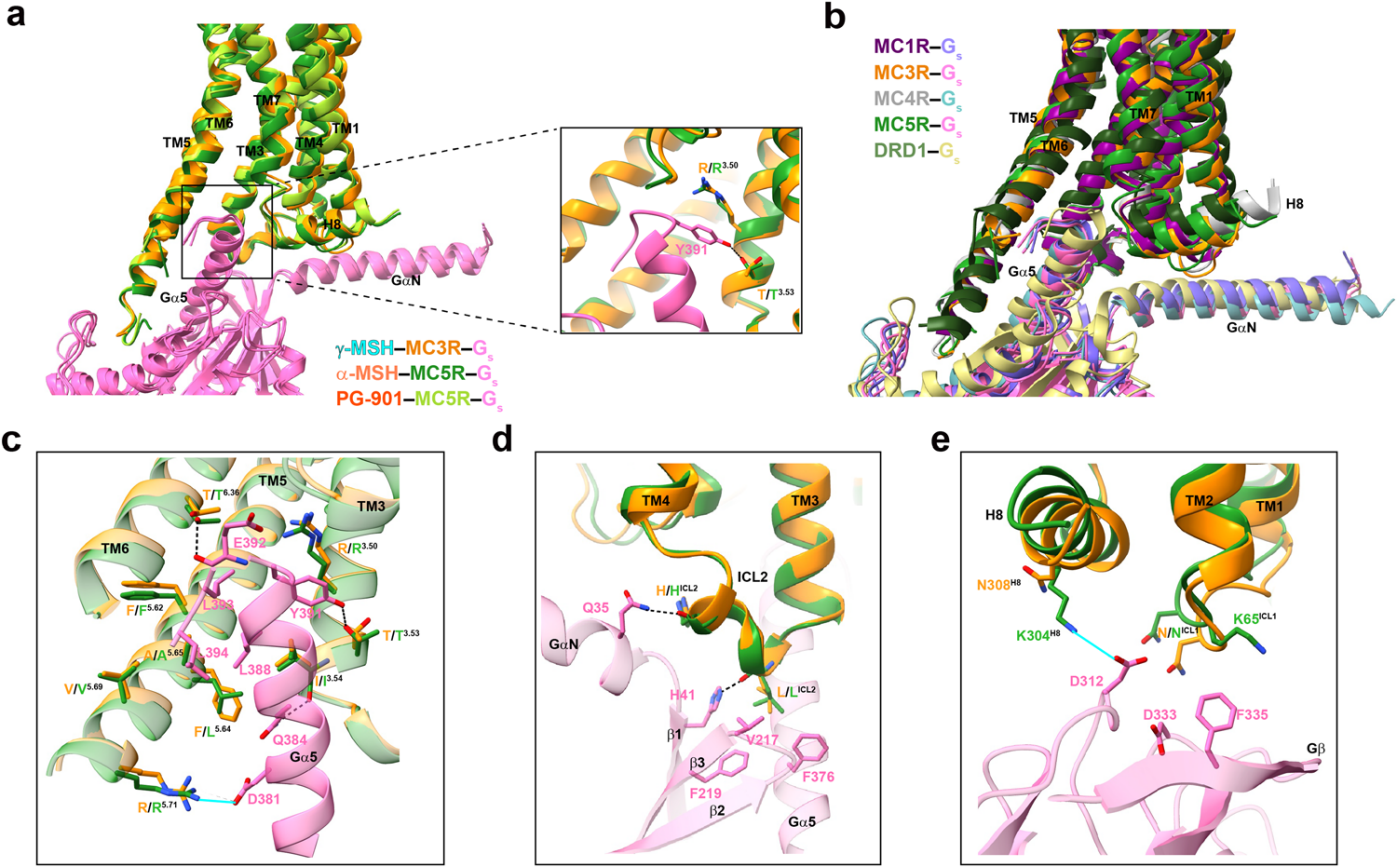
G protein coupling of MC3R and MC5R. **a** G_s_ coupling is almost identical among γ-MSH-bound MC3R, α-MSH-bound and PG-901-bound MC5R structures. The alignment is based on the receptor. **b** Comparison of G protein coupling among MC1R (PDB code: 7F4D), MC3R, MC4R (PDB code: 7F53), MC5R and DRD1 (PDB code: 7CKW). The receptors and G proteins were colored as labeled. **c** Interactions between MC3R (dark orange)/MC5R (forest green) and α5 helix of Gα_s_ in the cavity of the cytoplasmic region. **d** Interactions between ICL2 of both receptors and G_s_. **e** Interactions between helix 8/ICL1 and G_s_. Hydrogen bonds and salt bridges are shown as black dashed lines and cyan solid lines, respectively.

G_s_ protein coupling was almost identical among the three complex structures (Fig. 7a). Structural comparison of MC3R–G_s_ and MC5R–G_s_ complexes with other class A GPCRs demonstrates a high degree of similarity and suggests that MC3R and MC5R follow common G protein coupling mechanisms^39^, consistent with that observed in MC1R (PDB code: 7F4D)^27^, MC4R (PDB code: 7F53)^26^ and dopamine receptor D1 (DRD1, PDB code: 7CKW)^40^ (Fig. 7b). The Gα_s_ protein was anchored by the α5 helix, which fitted to the cytoplasmic cavity formed by TM3, TM4, TM5–TM7 and ICL1–ICL3. The C-terminus of G_s_ formed several polar interactions including three hydrogen bonds (T^6.36^‒E392, I^3.54^‒Q384 and T^3.53^‒Y391) and one salt bridge (R^5.71^‒D381), and hydrophobic contacts with the hydrophobic residues in TM3 and TM5 (I^3.54^, F^5.62^, L/F^5.64^, A^5.65^ and V^5.69^) via its hydrophobic patch (L388, L393 and L394). Meanwhile, several MCR-specific structural features were observed. One hydrogen bond between the side chain of Y391 in Gα_s_ and T^3.53^ in MCRs was found to be important for G_s_ coupling^26,27^ (Fig. 7a, c). Similar to ICL2 in MC1R and MC4R, ICL2 of MC3R and MC5R both adopted a short α-helix conformation and inserted into a pocket formed by αN–β1 hinge, β2–β3 loop and α5 helix of Gα_s_, resulting in profound ICL2–G protein interfaces (802 Å^2^ for MC3R and 671 Å^2^ for MC5R) and two hydrogen bonds (L150^ICL2^(MC3R)/L148^ICL2^(MC5R)–H41, H153^ICL2^(MC3R)/H151^ICL2^(MC5R)–Q35) (Fig. 7d). Besides, a salt bridge was formed between helix 8 of MC5R (K304) and Gβ subunit (D312), and this was not observed in MC3R (Fig. 7e).

## Discussion

MCRs belong to the peptide-binding receptor subfamily of class A GPCRs, and the five members (MC1R–MC5R) are promising drug targets for the treatment of multiple diseases. In this study, we report three high-resolution cryo-EM structures: γ-MSH-bound MC3R, α-MSH-bound and PG-901-bound MC5R in complex with G_s_ protein. Together with MC1R, MC2R and MC4R, whose structures have been determined recently, all MCRs have a short ECL2 and helical ECL3, thereby providing a wide and open orthosteric pocket in the extracellular side to accommodate their endogenous peptides. All the three bound peptides in this study adopt a “U-shape” conformation with the middle region (X–X–R–W motif) being the deepest segment within the receptor core^26–29,38^. Such a peptide binding mode is distinct from those observed in other class A GPCRs including relaxin family peptide receptor 4 (RXPF4)^39^, C-X-C motif chemokine receptor 2 (CXCR2)^41^ and cholecystokinin A receptor (CCKAR)^42^ and also class B1 GPCRs including glucagon-like peptide-1 receptor (GLP-1R)^43^, glucose-dependent insulinotropic polypeptide receptor (GIPR)^44^ and glucagon receptor (GCGR)^45^. Combined with mutagenesis experiments, these structures provide molecular basis of ligand recognition, subtype selectivity and divalent ion modulation of MC3R and MC5R, and will certainly be of value for the design of better therapeutics with desired pharmacological profiles.

MCRs differ in tissue distribution and physiological functions^46^. MC3R is expressed mostly in the hypothalamus and is responsible for energy homeostasis and immunomodulation^16,47,48^. A pervious study reported that γ-MSH has a higher potency for MC3R than for other MCRs^49^, consistent with our functional assay results (Fig. 3f). Combined with mutagenesis and structure/sequence analysis, the γ-MSH–MC3R complex structure highlights that the C-terminus of γ-MSH and the surrounding receptor residues, especially I265^6.58^, serve as structural determinants of subtype selectivity. For MC5R, both common and unique aspects of α-MSH (endogenous agonist) and PG-901 (synthetic cyclic peptide with high potency) recognition were illustrated. First, the recognition pattern of α-MSH is highly consistent across MC5R, MC1R^27^ and MC4R^26^. Second, PG-901, a shorter peptide but displays equal potency as α-MSH, successfully maintains massive MC5R interactions by expanding the hydrophobic and stacking contacts at DNal(2’)^7^ (F^7^ in α-MSH) to compensate the energy loss at P^6^ (H^6^ in α-MSH). Besides, the interaction between DNal(2’)^7^ of PG-901 and V^3.36^ (V126^3.36^ in MC5R and L128^3.36^ in MC3R) is likely responsible for the peptidic agonism at WT MC5R and MC3R mutant L128V but antagonism at WT MC3R. Third, the cyclization of PG-901 at D^5^ and K^10^ mimics the salt bridge (E^5^–K^11^) by connecting the two termini of α-MSH and reducing the entropy cost during peptide binding, thereby favoring the agonist potency. Meanwhile, binding of Ca^2+^ is mainly stabilized by the conserved EDD motif (E^2.64^, D^3.25^ and D^3.29^) in MCRs and the backbones of melanocortin peptides. Substitutions of these receptor residues by either alanine or asparagine/glutamine decreased the potency, efficacy and affinity to various extents. In a previously reported SHU9119–MC4R complex structure^39^, Ca^2+^ was observed as a co-factor and found to increase the affinity and potency of the endogenous agonist α-MSH at MC4R. The presence of Ca^2+^ preferentially increased the γ-MSH-induced cAMP accumulation at MC3R/MC1R and ACTH-induced cAMP accumulation at MC2R, compared to that of α-MSH at MC5R, suggesting that MC1R, MC2R and MC3R were more likely to be stabilized by Ca^2+^. Notably, in contrast to the agonism effect of Zn^2+^ at MC1R^36^, Zn^2+^ inhibited the efficacy of the three peptides investigated in this work at MC3R or MC5R. Similar but weaker inhibitory effect was observed with Fe^2+^. Mg^2+^ has little impact on G_s_ signaling mediated by MC3R and MC5R. These results demonstrate diverse and receptor-dependent modulatory roles of divalent ions in MCR-mediated signal transduction.

Together with the previously reported full-length structures of MC1R, MC2R and MC4R, the newly determined structures of MC3R and MC5R not only provide additional information of this important GPCR subfamily, but also allow us to analyze their commonality and uniqueness in a comprehensive manner. This will certainly advance drug discovery endeavor targeting MCRs for treatment of multiple diseases.

## Materials and methods

### Constructs

The full-length human MC3R and MC5R were cloned into pFastBac vector (Invitrogen) with haemagglutinin (HA) signal peptide at the N-terminus to enhance receptor expression. To obtain complexes with good homogeneity and stability, we used the NanoBiT tethering strategy, in which the C-terminus of human Gβ1 was linked to the HiBiT subunit (peptide 86, Promega) with a 15-amino acid polypeptide (GSSGGGGSGGGGSSG) linker and LgBiT subunit (Promega) was fused at the C-terminus of MC3R or MC5R connected by the 15-amino acid linker. A TEV protease cleavage site and double maltose-binding protein (2MBP) tag were added after LgBiT^50^. An engineered G_s_ construct (G112) designed based on mini-G_s_ as previously described^51,52^ was used to stabilize γ-MSH-bound MC3R or α-MSH- and PG-901-bound MC5R. The constructs were cloned into both pcDNA3.1 and pFastBac vectors for functional assays in mammalian cells and protein expression in insect cells, respectively. Other constructs including the WT MCRs and MCR mutants were cloned into pcDNA3.1 vector for cAMP accumulation and whole-cell binding assays. The human Gβ1 with a 15-amino acid linker followed by a HiBiT (peptide 86, Promega) and the bovine Gγ2 were cloned into pFastBac vector, respectively.

### Preparation of Nb35

Nanobody 35 (Nb35) with a C-terminal 6× His tag was expressed in the periplasm of *E. coli* BL21 (DE3) cells and cultured in TB medium (supplemented with 100 μg/mL ampicillin, 2 mM MgCl_2_, 0.1% (w/v) glucose) at 37 °C, 180 rpm. When OD_600_ value reached 0.7–1.2, 1 mM IPTG was added to induce expression followed by overnight incubation at 28 °C. Cells were harvested by centrifugation (2000 rpm, 20 min) and stored at –80 °C. Nb35 was purified by size-exclusion chromatography using a HiLoad 16/600 Superdex 75 column (GE Healthcare) with running buffer containing 20 mM HEPES and 100 mM NaCl, pH 7.4. The monomeric fractions supplemented with 30% (v/v) glycerol were flash-frozen in liquid nitrogen and stored at –80 °C.

### Complex formation and purification

The MCR (MC3R or MC5R), engineered Gα_s_, Gβ1 and Gγ2 constructs were co-expressed in *Spodoptera frugigiperda* (*Sf*9) insect cells using the Bac-to-Bac Baculovirus Expression System (Invitrogen). Cells were transfected with the above four baculoviruses with a ratio of 1:1:1:1 at a density of 3.0 × 10^6^ cells/mL. The cells were cultured for 48 h at 27 °C after infection and collected by centrifugation at 2000 rpm for 20 min.

For the α-MSH–MC5R–G_s_ complex, the frozen cell pellets from 1 L culture were thawed on ice and resuspended in the lysis buffer (20 mM HEPES, pH 7.4, 100 mM NaCl and 10% (v/v) glycerol supplemented with EDTA-free protease inhibitor cocktail (Bimake)). Cells were lysed by dounce homogenization and complex formation was initiated with addition of 10 μM α-MSH (GL Biochem), 10 μg/mL Nb35, 25 mU/mL apyrase (NEB), 10 mM MgCl_2_, 1 mM MnCl_2_ and 250 μM TCEP, supplemented with EDTA-free protease inhibitor cocktail (Bimake) for 1.5 h incubation at room temperature (RT). The membrane was then solubilized with 0.5% (w/v) lauryl maltose neopentyl glycol (LMNG, Anatrace) and 0.1% (w/v) cholesterol hemisuccinate (CHS, Anatrace) with additional protease inhibitor cocktail for 3 h at 4 °C. The supernatant was isolated by centrifugation at 32,000 rpm for 45 min and incubated with amylose resin (NEB) for 2 h at 4 °C. The resin was collected and packed into a gravity flow column and washed with 20 column volumes of wash buffer (5 μM γ-MSH, 0.01% (w/v) LMNG, 0.002% (w/v) CHS, 0.01% (w/v) GDN, 0.008% (w/v) CHS, 20 mM HEPES, pH 7.4, 100 mM NaCl, 10% (v/v) glycerol, 2 mM MgCl_2_, 2 mM MnCl_2_ and 25 μM TCEP). 2MBP tag was removed by His-tagged TEV protease (customer-made) during overnight incubation. The complexes were concentrated using a 100-kD Amicon Ultra centrifugal filter (Millipore) and subjected to Superose6 increase 10/300 GL column (GE Healthcare) with running buffer containing 20 mM HEPES, pH 7.4, 100 mM NaCl, 2 mM MgCl_2_, 250 μM TCEP, 5 μM γ-MSH, 0.00075% (w/v) LMNG, 0.00025% (w/v) GDN, 0.00025% digitonin and 0.0002% (w/v) CHS. The monomeric peak fractions were pooled and concentrated to 4 mg/mL. All procedures mentioned above were performed at 4 °C.

The purification processes of the γ-MSH–MC3R–G_s_ and PG-901–MC5R–G_s_ complexes were similar to that of the α-MSH–MC5R–G_s_ complex, except that α-MSH was replaced by γ-MSH and PG-901. The complex samples were concentrated to 5 mg/mL and 8 mg/mL for the cryo-EM analysis of γ-MSH–MC3R–G_s_ and PG-901–MC5R–G_s_, respectively.

### Cryo-EM data acquisition

For preparation of cryo-EM grids, 3 μL of freshly purified complex samples were applied to glow-discharged holey grids (Quantifoil R1.2/1.3, 300 mesh) and subsequently vitrified using a Vitrobot Mark IV (Thermo Fisher Scientific) set at 100% humidity and 4 °C. Cryo-EM images were collected on a Titan Krios microscope (FEI) equipped with Gatan energy filter, K3 direct electron detector and serial EM3.7. The microscope was operated at 300 kV accelerating voltage, at a nominal magnification of 46,685× in counting mode, corresponding to a pixel size of 1.071 Å. Totally, 5198 movies of the γ-MSH–MC3R–G_s_ complex, 5078 movies of the α-MSH–MC5R–G_s_ complex and 5156 movies of the PG-901–MC5R–G_s_ complex were obtained with a defocus range of –1.2 μm to –2.2 μm. An accumulated dose of 80 electrons per Å^2^ was fractionated into a movie stack of 36 frames.

### Cryo-EM data processing

Dose-fractionated image stacks were subjected to beam-induced motion correction using MotionCor2.1^53^. A sum of all frames, filtered according to the exposure dose, in each image stack was used for further processing. Contrast transfer function parameters for each micrograph were determined by Gctf v1.06^54^. Particle selection, two-dimensional (2D) and 3D classifications, were performed on a binned dataset with a pixel size of 2.142 Å using cryoSPARC v3.2.0 and RELION-3.1.1.

For the dataset of the γ-MSH–MC3R–G_s_ complex, particle picking was done with “Blob picker” using a particle diameter of 180 Å. Particle images were extracted with a box size of 256 pixel. After reference-free 2D classification, selected class averages were used for template-based particle picking. A total of 3,680,575 particle projections were subjected to two rounds of 2D classification to discard false positive particles or particles categorized in poorly defined classes, producing 1,497,042 particle projections for further processing. A 3D reference model of particle images was generated by ab initio reconstruction, and heterogeneous refinement was applied to divide particles into three subsets. A selected subset containing 1,255,719 particles was subjected to further 3D auto-refinement with a mask on the complex followed by a new round of 3D classification with a mask on the G protein. A dataset of 1,102,647 particles was subjected to 3D refinement, yielding a final map with a global nominal resolution of 2.86 Å by the 0.143 criteria of the gold-standard Fourier shell correlation (FSC). Half-reconstructions were used to determine the local resolution of each map.

The single-particle cryo-EM analysis of the α-MSH–MC5R–G_s_ and PG-901–MC5R–G_s_ complexes were similar to that of the γ-MSH–MC3R–G_s_ complex as shown in Supplementary Fig. S2 and Table S1. For the α-MSH–MC5R–G_s_ complex, a dataset of 568,095 particles was subjected to 3D refinement, yielding a final map with a global nominal resolution of 2.73 Å. After the last round of refinement, the final map of PG-901–MC5R–G_s_ complex has an indicated global resolution of 2.59 Å at an FSC of 0.143, based on a dataset of 803,492 particles and following 3D refinement. Half-reconstructions were used to determine the local resolution of each map.

### Model building and refinement

The initial model of MC5R–G_s_ complex was built using the cryo-EM structure of α-MSH–MC4R–G_s_ complex (PDB code: 7F53)^26^, and LY3298176–GIPR–G_s_ (PDB code: 7VAB)^55^ was applied to G protein modeling. The initial model of MC3R–G_s_ complex was built using the cryo-EM structure of α-MSH–MC1R–G_s_ complex (PDB code: 7F4D)^27^. The initial models were docked into the EM density map using UCSF Chimera v1.13.1^56^, followed by iterative manual adjustment and rebuilding in COOT 0.9.8.1^57^. The CheckMyMetal (CMM) webserver was used to validate and analyze Ca^2+^ assignment in the structure model^58^. Real-space refinement was performed using PHENIX v1.16^59^. The final refinement statistics were validated using the module comprehensive validation (cryo-EM) in PHENIX^60^. Structural figures were prepared in UCSF Chimera v1.13.1, UCSF ChimeraX v1.0 and PyMOL v.2.1 (https://pymol.org/2/). The final refinement statistics are provided in Supplementary Table S1.

### cAMP accumulation assay

WT or mutant MCR constructs were cloned into pcDNA3.1 vector (Invitrogen) for functional studies. HEK293T cells were transiently transfected with the vectors using Lipofectamine 2000 transfection reagent (Invitrogen) and incubated at 37 °C in 5% CO_2_. Twenty-four hours after transfection, the transfected cells were digested with 0.02% (w/v) EDTA, resuspended in stimulation buffer (Hanks’ balanced salt solution (HBSS)) supplemented with 5 mM HEPES, 0.5 mM IBMX and 0.1% (w/v) bovine serum albumin (BSA), pH 7.4) to a density of 6 × 10^5^ cells/mL and added to 384-well white plates (PerkinElmer, 3000 cells per well). cAMP accumulation was measured by a LANCE Ultra cAMP kit (PerkinElmer) according to the manufacturer’s instructions. In brief, transfected cells were incubated for 40 min in stimulation buffer with different concentrations of ligands (5 μL) at RT. The reaction was stopped by addition of lysis buffer containing 5 μL Eu-cAMP tracer and 5 μL ULight-anti-cAMP. Plates were then incubated for 50 min at RT and time-resolved FRET signals were measured at 620 nm and 665 nm, respectively, by an EnVision multilabel plate reader (PerkinElmer). Data were analyzed in GraphPad PRISM 8 and all values were normalized to the WT.

For assessing the effect of divalent ions on cAMP signaling, HEK293T cells were digested with 0.02% (w/v) EDTA and washed three times with calcium/magnesium-free HBSS buffer. Then the cells were resuspended and stimulated with different concentrations of ligands in Ca^2+^-free stimulation buffer (the aforementioned stimulation buffer supplemented with 1 mM EGTA), or with additional 2 mM divalent ions in Ca^2+^-free stimulation buffer ([divalent ions] = ~1 mM)^38^. The rest of steps were essentially the same as described above.

### Whole-cell binding assay

HEK293 cells were cultured in DMEM medium with 10% FBS and seeded at a density of 30,000 cells/well in Isoplate-96 plates (PerkinElmer). Twenty-four hours after transfection with WT or mutant constructs, cells were washed twice and incubated with blocking buffer (DMEM supplemented with 25 mM HEPES and 0.1% (w/v) BSA, pH 7.4) for 2 h at 37 °C. For homogeneous competition binding, radiolabeled [^125^I]-[Nle^4^,d-Phe^7^]-α-MSH (30 pM, PerkinElmer) and seven decreasing concentrations of unlabeled peptide (γ-MSH (50 μM to 128 pM) or α-MSH (1 μM to 4 pM) or PG-901 (1 μM to 4 pM)) were added and competitively reacted with the cells in blocking buffer at RT for 3 h. Following incubation, cells were washed three times with ice-cold PBS and lysed by 50 μL lysis buffer (PBS supplemented with 20 mM Tris-HCl, 1% Triton X-100, pH 7.4). The radioactivity was subsequently counted (counts per minute, CPM) in a scintillation counter (MicroBeta^2^ Plate Counter, PerkinElmer) using a scintillation cocktail (OptiPhaseSuperMix, PerkinElmer).

### Receptor surface expression

Cell surface expression was determined by flow cytometry of the N-terminal Flag tag on the WT and mutant MC3R or MC5R transiently expressed in HEK293T cells. All the mutant constructs were modified by single-point mutation in the setting of the WT construct. Briefly, ~3 × 10^5^ cells were blocked with PBS containing 5% BSA (w/v) at RT for 15 min and incubated with 1:300 anti-Flag primary antibody (diluted with PBS containing 5% BSA, Sigma-Aldrich) at RT for 1 h. The cells were then washed three times with PBS containing 1% BSA (w/v) followed by 1 h incubation with 1:1000 anti-mouse Alexa Fluor 488-conjugated secondary antibody (diluted with PBS containing 5% BSA, Invitrogen) at 4 °C in the dark. After washing three times, cells were resuspended in 200 μL PBS containing 1% BSA for detection by Flow Cytometer (BD Biosciences) utilizing laser excitation and emission wavelengths of 488 nm and 530 nm, respectively. For each sample, 10,000 cellular events were collected, and the total fluorescence intensity of positive expression cell population was calculated by NovoExpress 1.2.1 software (Agilent). Data were normalized to the WT receptor.

## Supplementary information

Supplementary Information

## Data Availability

The cryo-EM density maps have been deposited in the Electron Microscopy Data Bank (EMDB) under accession codes EMD-35615 (γ-MSH–MC3R–G_s_ complex), EMD-35601 (α-MSH–MC5R–G_s_ complex) and EMD-35616 (PG-901–MC5R–G_s_ complex). Coordinates have been deposited in the Protein Data Bank (PDB) under accession codes 8IOC (γ-MSH–MC3R–G_s_ complex), 8INR (α-MSH–MC5R–G_s_ complex) and 8IOD (PG-901–MC5R–G_s_ complex).
