# Supplementary material for: Structural insights into ligand recognition and subtype selectivity of the human melanocortin-3 and melanocortin-5 receptors
Source: Cell Discov. 2023 Jul 31;9:81. doi: 10.1038/s41421-023-00586-4 (PMC10390531; doi:10.1038/s41421-023-00586-4)
Supplement: Supplementary file 1 — Supplementary Information [file 41421_2023_586_MOESM1_ESM.pdf]

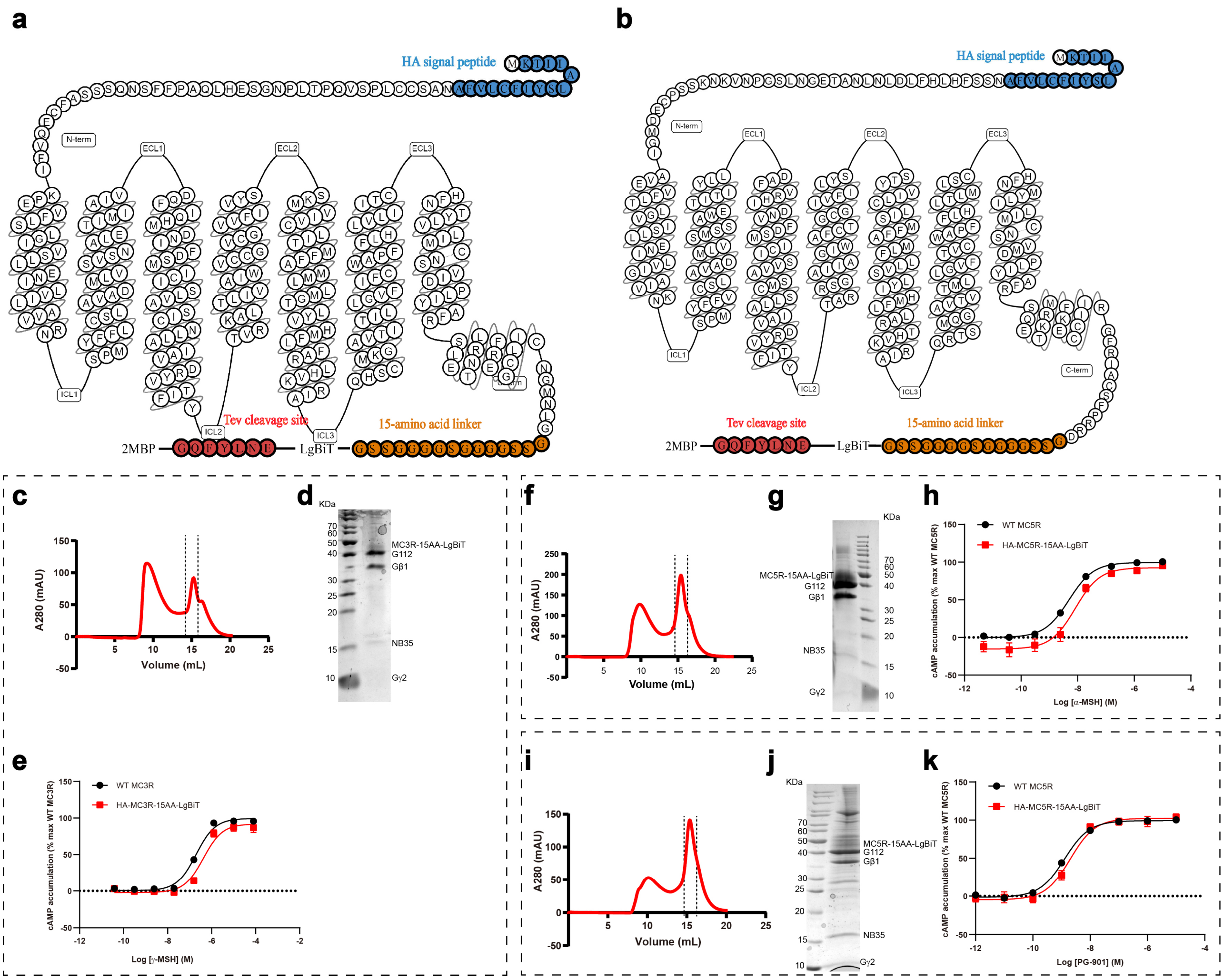

Supplementary Figure S1. Purification and characterization of the γ-MSH–MC3R–G$_s$–Nb35, α-MSH–MC5R–G$_s$–Nb35 and PG-901–MC5R–G$_s$–Nb35 complexes. a Snake-plot diagram of the human MC3R–LgBiT construct HA–MC3R–15AA–LgBiT–2MBP. The resultant γ-MSH–MC3R–G$_s$ complex structure was determined by cryo-EM at 2.86 Å. **b** Snake-plot diagram of the human HA–MC5R–15AA–LgBiT–2MBP. The resultant α-MSH-bound and PG-901-bound–MC5R–G$_s$ complex structure was determined by cryo-EM at 2.73 Å and 2.59 Å, respectively. **c-d** Analytical size-exclusion chromatography of the γ-MSH–MC3R–G$_s$ (**c**), SDS-PAGE/Coomassie blue stain **(d)** of the purified γ-MSH–MC3R–G$_s$ complex. **e** cAMP responses following γ-MSH stimulation in HEK293T cells transfected with wildtype (WT) or modified MC3R construct. **f-g** Analytical size-exclusion chromatography of the α-MSH–MC5R–G$_s$ (**f**), SDS-PAGE/Coomassie blue stain **(g),** of the purified α-MSH–MC5R–G$_s$ complex. **h** cAMP responses following α-MSH stimulation in HEK293T cells transfected with WT or modified MC5R construct. **i-j** Analytical size-exclusion chromatography of the PG-901–MC5R–G$_s$ (**i**), SDS-PAGE/Coomassie blue stain **(j)** of the purified PG-901–MC5R–G$_s$ complex. **k** cAMP responses following PG-901 stimulation in HEK293T cells transfected with WT or modified MC5R construct.

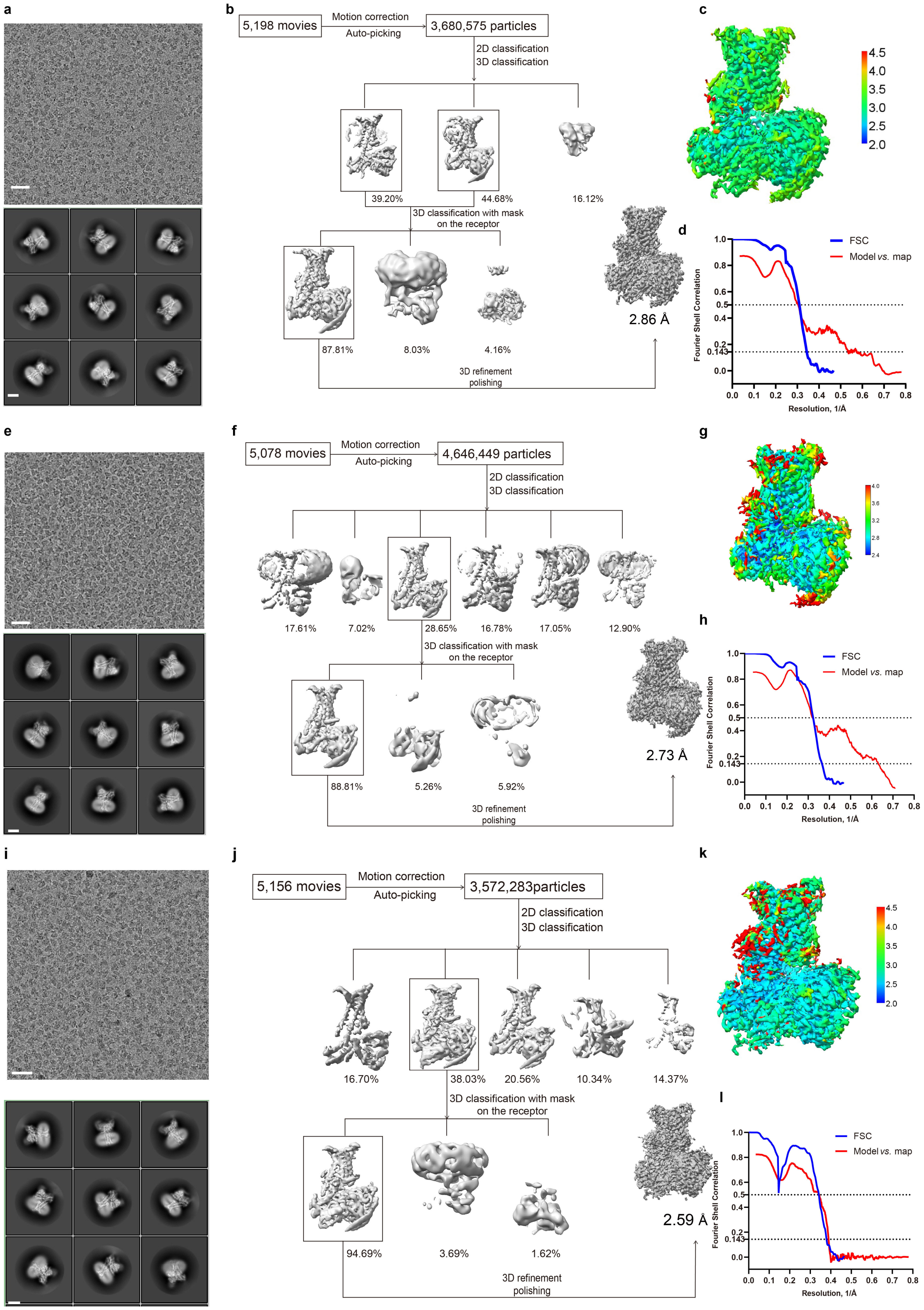

Supplementary Figure S2. Cryo-EM analysis of the γ-MSH–MC3R–G$_s$, α-MSH–MC5R–G$_s$ and PG-901–MC5R–G$_s$ complexes. a-d Workflow of the γ-MSH–MC3R–G$_s$ complex. **a** Representative cryo-EM micrograph

(scale bar: 40 nm) and two-dimensional class averages (scale bar: 5 nm) of. **b** Flowchart of cryo-EM data processing. **c** Local resolution distribution map. **d** Gold-standard Fourier shell correlation (FSC) curves of the map (blue) and the model *vs*. map (red). **e-h** Workflow of the α-MSH–MC5R–G_s complex. **e** Representative cryo-EM micrograph (scale bar: 40 nm) and two-dimensional class averages (scale bar: 5 nm). **f** Flowchart of cryo-EM data processing. **g** Local resolution distribution map. **h** Gold-standard FSC curves of the map (blue) and the model *vs*. map (red). **i-l** Workflow of the PG-901–MC5R–G_s complex. **i** Representative cryo-EM micrograph (scale bar: 40 nm) and two-dimensional class averages (scale bar: 5 nm). **j** Flowchart of cryo-EM data processing. **k** Local resolution distribution map. **l** Gold-standard FSC curves of the map (blue) and the model *vs*. map (red).

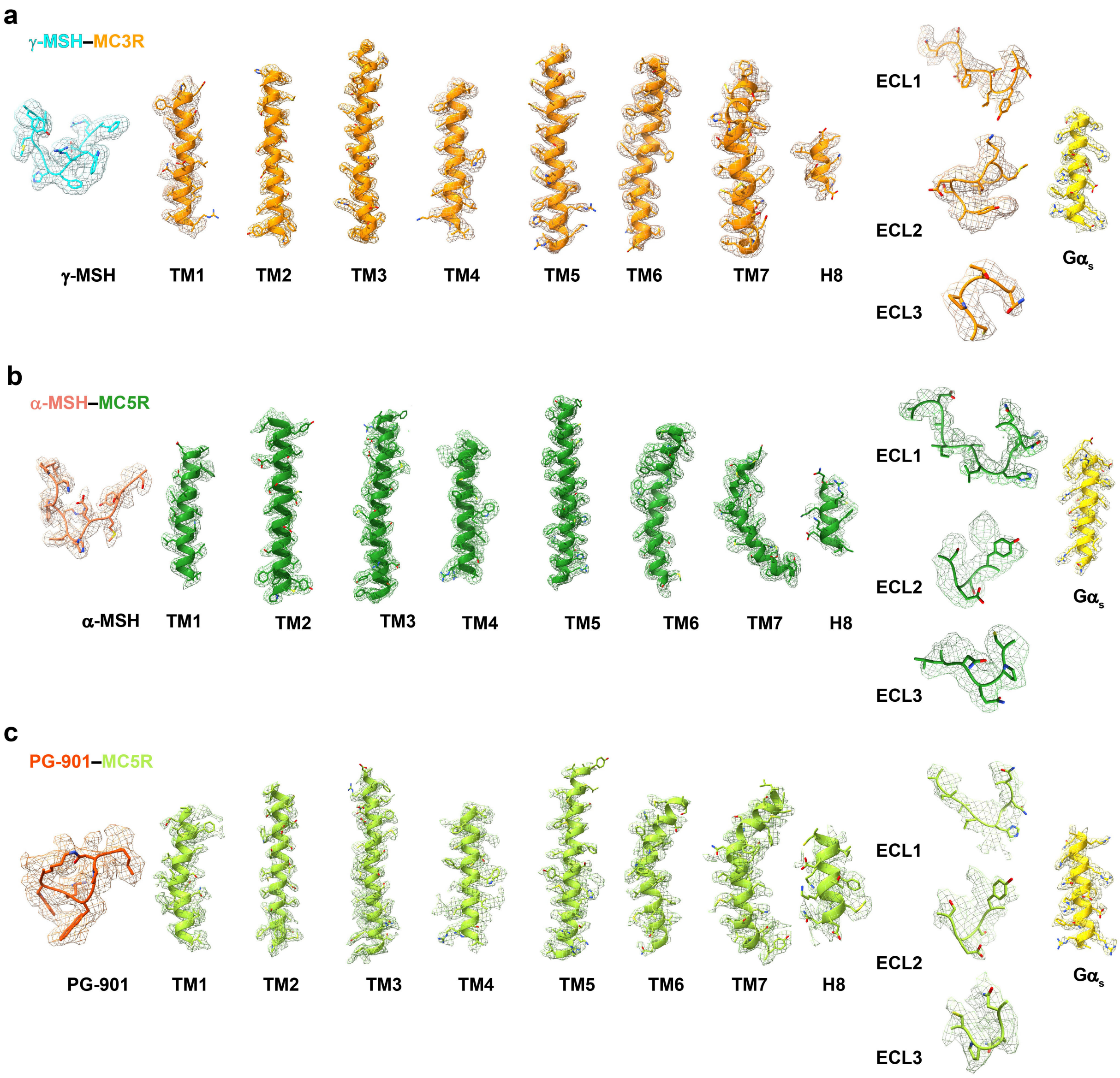

Supplementary Figure S3. Atomic resolution models of the γ-MSH–MC3R–G_s, α-MSH–MC5R–G_s, and PG-901–MC5R–G_s complexes in the cryo-EM density maps. a EM density map and model of the γ-MSH–MC3R–G_s are shown for all seven transmembrane α-helices, helix 8 (H8) and all extracellular loops of MC3R, the α5-helix (H5) of the $G\alpha_s$ Ras-like domain and γ-MSH. **b** EM density map and model of the α-MSH–MC5R–G_s are shown for all seven transmembrane α-helices, H8 and all extracellular loops of MC5R, the H5 of the $G\alpha_s$ Ras-like domain and α-MSH. **c** EM density map and model of the PG-901–MC5R–G_s are shown for all seven transmembrane α-helices, H8 and all extracellular loops of MC5R, the H5 of the $G\alpha_s$ Ras-like domain and PG-901.

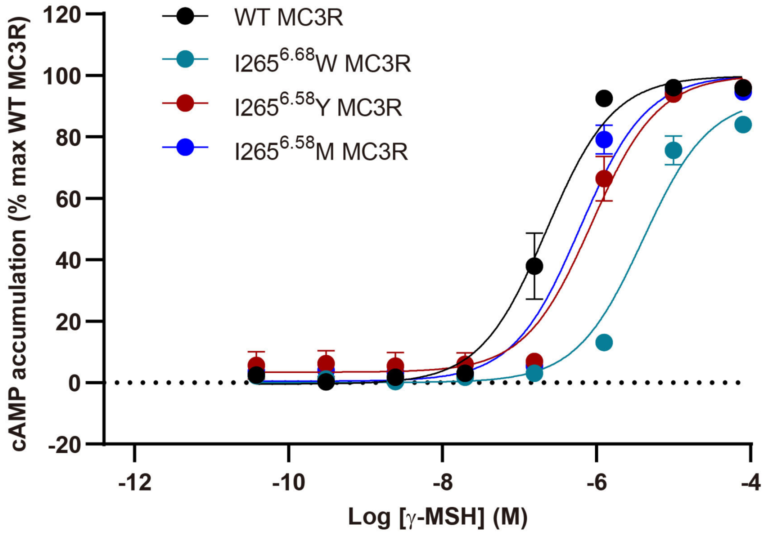

Supplementary Figure S4. Effects of MC3R I265$^{6.58}$ mutation on γ-MSH-induced cAMP accumulation. Data shown are from at least three independent experiments performed in quadruplicate.

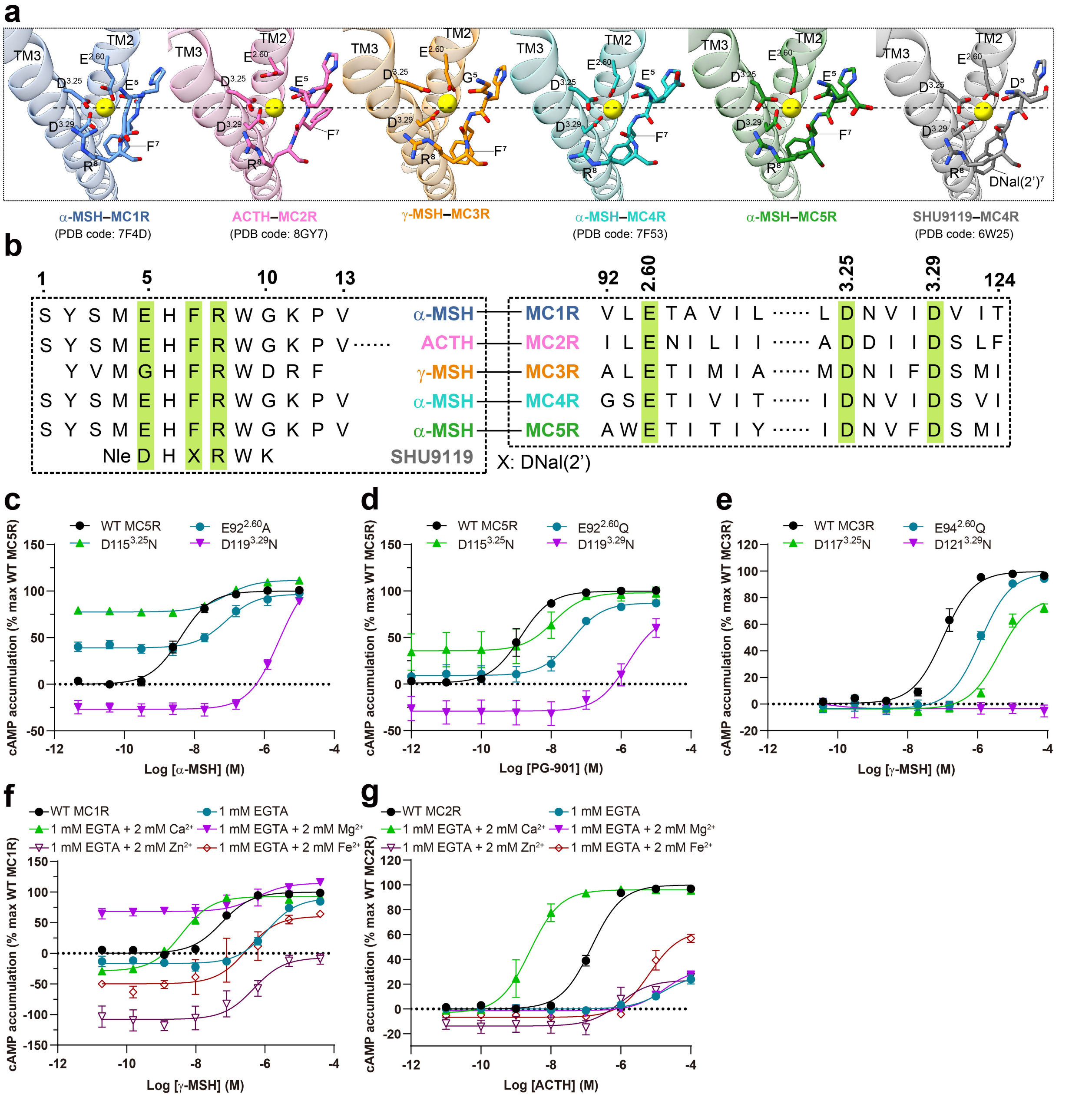

Supplementary Figure S5. Structural comparison and functional assessment of the calcium ion-binding pocket in MCRs. a Conformational comparison of the calcium ion-binding pocket among MCRs. All structures are superimposed on the α-MSH–MC1R (PDB code:7F4D) using the Cα carbons of the TMD residues. The calcium is shown in yellow sphere. The location of Ca^{2+} in α-MSH–MC1R is shown as black dashed lines for reference. **b** The sequence alignment of the calcium ion-binding pocket residues. Residues interacting with Ca^{2+} in MCRs and ligands are highlighted in lawn green. **c, d** Effects of MC5R mutations on α-MSH-induced **(c)** and PG-901-induced **(d)** cAMP accumulation. **e** Effects of MC3R mutations on γ-MSH-induced cAMP response. **f** Effects of divalent ions on γ-MSH-stimulated cAMP response of MC1R. **g** Effects of divalent ions on ACTH-stimulated cAMP response of MC2R. Data shown are from at least three independent experiments performed in quadruplicate.

Supplementary Table S1. Cryo-EM data collection, refinement and validation statistics.

	γ-MSH–MC3R–G_s–Nb35 complex	α-MSH–MC5R–G_s–Nb35 complex	PG-901–MC5R–G_s–Nb35 complex
**Data collection and processing**			
Magnification	46,685	46,685	46,685
Voltage (kV)	300	300	300
Electron exposure (e^-/Å^2)	80	80	80
Defocus range (μm)	-1.2 to -2.2	-1.2 to -2.2	-1.2 to -2.2
Pixel size (Å)	1.071	1.071	1.071
Symmetry imposed	Cl	C1	Cl
Initial particle images (no.)	3,680,575	4,646,449	3,572,283
Final particle images (no.)	1,102,647	568,095	803,492
Map resolution (Å)	2.86	2.73	2.59
FSC threshold	0.143	0.143	0.143
Map resolution range (Å)	2.0-4.5	2.4-4.0	2.0-4.5
**Refinement**			
Initial model used (PDB code)	7F4D	7F53, 7VAB	7F53, 7VAB
Model resolution (Å)	3.3	3.2	2.9
FSC threshold	0.5	0.5	0.5
Model resolution range (Å)	2.0-4.5	3.0-5.0	2.0-4.5
Map sharpening B factor (Å^2)	-121.9	-103.6	-114.0
Model composition			
Non-hydrogen atoms	8,223	7,220	8,062
Protein residues	1,041	912	1,022
Lipids	0.00	0.00	0.00
B factors (Å^2)			
Protein	119.32	141.00	94.32
Ligand	60.00	45.00	33.16
R.m.s. deviations			
Bond lengths (Å)	0.007	0.012	0.004
Bond angles (°)	0.704	0.852	0.780
Validation			
MolProbity score	1.91	1.91	1.98
Clash score	9.59	11.46	13.47
Poor rotamers (%)	0.00	0.00	0.00
Ramachandran plot			
Favored (%)	93.95	95.10	95.00
Allowed (%)	6.05	4.90	5.00
Disallowed (%)	0.00	0.00	0.00

Supplementary Table S2. Interaction between γ-MSH and MC3R in the γ-MSH–MC3R–G_s complex structure.

γ-MSH	MC3R	Interactions
Y2	$D110^{3.18}$	Hydrogen bonds
	$I113^{3.21}/Q114^{3.22}$	Hydrophobic interactions
M4	$M97^{2.63}/I98^{2.64}/I113^{3.21}/D117^{3.25}$	Hydrophobic interactions
G5	$I98^{2.64}$	Hydrophobic interactions
H6	$F45^{1.39}$	Pi-Pi stacking
	$T95^{2.61}/N282^{7.36}$	Hydrogen bonds
	$T95^{2.61}/I98^{2.64}/L285^{7.39}$	Hydrophobic interactions
F7	$I124^{3.32}/C125^{3.33}/L128^{3.36}/F258^{6.51}/F281^{7.35}/L285^{7.39}$	Hydrophobic interactions
R8	$D121^{3.29}$	Salt bridges
	$N118^{3.26}/I180^{4.61}/S183^{ECL2}$	Hydrogen bonds
	$I180^{4.61}$	Hydrophobic interactions
W9	$S183^{ECL2}/H261^{6.54}$	Hydrogen bonds
	$C125^{3.33}/I180^{4.61}/V188^{5.39}/I189^{5.40}/L192^{5.43}/L262^{6.65}$	Hydrophobic interactions
D10	$Q37^{N\text{-}term}$	Hydrogen bonds
	$F281^{7.35}$	Hydrophobic interactions
F12	$E184^{ECL2}/K186^{5.37}/I189^{5.40}/I265^{6.58}$	Hydrophobic interactions

Superscripts refer to the Ballesteros-Weinstein numbers and residues with 4 Å were shown[1].

Supplementary Table S3. Interaction between α-MSH and MC5R in the α-MSH–MC5R–G_s complex structure.

α-MSH	MC5R	Interactions
S1	D108$^{3.18}$	Hydrogen bonds
Y2	D115$^{3.25}$	Hydrogen bonds
	V111$^{3.21}$/R112$^{3.22}$	Hydrophobic interactions
S3	N100^{ECL1}	Hydrogen bonds
M4	E92$^{2.60}$/T95$^{2.63}$/I96$^{2.64}$/L99$^{2.67}$/V111$^{3.21}$/I114$^{3.24}$/F118$^{3.28}$	Hydrophobic interactions
E5	E92$^{2.60}$/I96$^{2.64}$	Hydrophobic interactions
H6	T93$^{2.61}$/N278$^{7.36}$	Hydrogen bonds
	F43$^{1.39}$	Pi-Pi interaction
	I96$^{2.64}$/N278$^{7.36}$/L281$^{7.39}$	Hydrophobic interactions
F7	E92$^{2.60}$/D119$^{3.29}$/I122$^{3.32}$/I178$^{4.61}$/F254$^{6.51}$/F277$^{7.35}$/L281$^{7.39}$	Hydrophobic interactions
R8	D115$^{3.25}$/D119$^{3.29}$	Salt bridges
	N116$^{3.26}$	Hydrogen bonds
	I178$^{4.61}$/S181$^{4.64}$	Hydrophobic interactions
W9	S181^{ECL2}	Hydrogen bonds
	V186$^{5.39}$/I187$^{5.40}$/L190$^{5.43}$/F254$^{6.51}$/L258$^{6.55}$/M261$^{6.58}$	Hydrophobic interactions
G10	H257$^{6.54}$	Hydrogen bonds
	F277$^{7.35}$	Hydrophobic interactions
K11	M261$^{6.58}$	Hydrophobic interactions
P12	M261$^{6.58}$/P265^{ECL3}/M274^{ECL3}	Hydrophobic interactions
V13	E182^{ECL2}	Hydrophobic interactions

Superscripts refer to the Ballesteros-Weinstein numbers and residues with 4 Å were shown[1].

Supplementary Table S4. Interaction between PG-901 and MC5R in the PG-901–MC5R–G_s complex structure.

PG-901	MC5R	Interactions
Nle4	$E92^{2.60}/T95^{2.63}/I96^{2.64}/D115^{3.25}/F118^{3.28}$	Hydrophobic interactions
D5	$I96^{2.64}$	Hydrophobic interactions
P6	$I96^{2.64}/L281^{7.39}$	Hydrophobic interactions
DNal(2')7	$F254^{6.51}/F255^{6.52}$	Pi-Pi Stacking
	$I122^{3.32}/V126^{3.36}/L190^{5.43}/L281^{7.39}$	Hydrophobic interactions
R8	$D115^{3.25}/D119^{3.29}$	Salt bridges
	$N116^{3.26}/I178^{4.61}/S181^{ECL2}$	Hydrogen bonds
	$I178^{4.61}/F277^{7.35}$	Hydrophobic interactions
W9	$S181^{ECL2}$	Hydrogen bonds
	$F177^{4.60}/I178^{4.61}/V186^{5.39}/I187^{5.40}/L190^{5.43}/L258^{6.55}/M261^{6.58}$	Hydrophobic interactions
K10	$F277^{7.35}$	Hydrophobic interactions

Superscripts refer to the Ballesteros-Weinstein numbers and residues with 4 Å were shown[1].

Supplementary Table S5. γ-MSH-induced cAMP responses at WT and mutant MC3Rs.

**Mutation**	**pEC_{50} ± S.E.M.**	**E_{max} ± S.E.M. (% WT)**	**Cell surface expression (% WT)**
WT MC3R	6.71 ± 0.04	100.00 ± 1.31	100.00
HA-MC3R(WT)-15AA-LgBiT	6.38 ± 0.10	92.42 ± 3.67	58.33 ± 4.42*
$Q37^{N\text{-}term}A$	6.70 ± 0.08	99.55 ± 2.71	91.60 ± 8.46
$F45^{1.39}A$	4.61 ± 0.28***	51.49 ± 9.70****	42.60 ± 5.23**
$E94^{2.60}A$	N.D.	N.D.	123.34 ± 12.78
$E94^{2.60}Q$	5.92 ± 0.09****	98.70 ± 3.77	91.07 ± 13.45
$I98^{2.64}A$	5.05 ± 0.10****	89.42 ± 5.36	85.72 ± 12.22
$D110^{3.18}A$	6.36 ± 0.09	101.00 ± 3.23	89.16 ± 7.07
$I113^{3.21}A$	6.27 ± 0.10*	101.66 ± 3.79	79.62 ± 12.00
$D117^{3.25}A$	5.18 ± 0.12****	44.58 ± 2.87****	95.66 ± 4.87
$D117^{3.25}N$	5.38 ± 0.12****	79.95 ± 4.89**	85.14 ± 10.04
$N118^{3.26}A$	6.81 ± 0.09	103.11 ± 3.12	98.35 ± 9.97
$D121^{3.29}A$	5.36 ± 0.11****	96.13 ± 5.12	87.94 ± 11.69
$D121^{3.29}N$	N.D.	N.D.	17.37 ± 2.62****
$I180^{4.61}A$	5.37 ± 0.11****	89.48 ± 4.65	71.86 ± 13.15
$S183^{ECL2}A$	6.42 ± 0.12	104.35 ± 4.30	96.01 ± 10.10
$K186^{5.37}A$	6.94 ± 0.05	102.31 ± 1.75	73.43 ± 7.34
$I189^{5.40}A$	6.11 ± 0.08***	103.90 ± 3.20	98.72 ± 14.37
$L192^{5.43}A$	6.15 ± 0.08**	105.05 ± 3.21	81.88 ± 12.24
$F258^{6.51}A$	5.24 ± 0.09****	82.83 ± 3.90*	34.13 ± 2.14***
$H261^{6.54}A$	5.21 ± 0.12****	64.39 ± 4.06****	45.91 ± 5.14**
$I265^{6.58}A$	6.22 ± 0.09**	101.54 ± 3.63	104.1 ± 13.10
$I265^{6.58}M$	6.22 ± 0.10**	100.16 ± 3.75	99.40 ± 11.61
$I265^{6.58}Y$	6.06 ± 0.10***	100.17 ± 3.84	73.28 ± 3.36
$I265^{6.58}W$	5.40 ± 0.09****	92.96 ± 4.06	31.34 ± 4.22***
$F281^{7.35}A$	6.27 ± 0.08*	106.02 ± 3.14	96.15 ± 12.71
$E94^{2.60}A/D117^{3.25}/D121^{3.29}A$	N.D.	N.D.	51.32 ± 6.25*

cAMP accumulation was normalized to the maximum response of wildtype (WT) MC3R and dose-response curves were analyzed using a three-parameter logistic equation to obtain pEC_{50} values. Cell surface expression was assessed by FACS and values were normalized to the WT (shown as percentage). The experiments were carried out independently at least three times. Data shown are means ± S.E.M. One-way ANOVA was used to determine statistical difference. N.D., not determined. *P< 0.05, **P< 0.01, ***P< 0.001 and ****P<0.0001.

Supplementary Table S6. Receptor binding profiles of γ-MSH at MC3R.

Mutation	pIC$_{50}$ ± S.E.M.	Span ± S.E.M. (% WT)
WT	7.04 ± 0.07	100.00 ± 3.47
Q37$^{N\text{-term}}$A	7.10 ± 0.14	102.93 ± 6.69
F45$^{1.39}$A	5.36 ± 0.57	N.D.
E94$^{2.60}$A	N.D.	N.D.
I98$^{2.64}$A	7.93 ± 0.59	11.43 ± 3.25****
D117$^{3.25}$A	5.85 ± 0.54	10.57 ± 3.34****
D121$^{3.29}$A	7.35 ± 0.52	11.74 ± 2.81****
I180$^{4.61}$A	7.64 ± 0.42	15.11 ± 2.95****
F258$^{6.51}$A	4.76 ± 0.54*	22.36 ± 13.55****
H261$^{6.54}$A	7.81 ± 0.69	14.79 ± 4.90****
E94$^{2.60}$A/D117$^{3.25}$A/D121$^{3.29}$A	N.D.	N.D.

Whole cell binding assay was performed in HEK293 cells. Binding data were analyzed using a three-parameter logistic equation to determine pIC$_{50}$ and span values. Data shown are means ± S.E.M. One-way ANOVA was used to determine statistical difference (*$P< 0.05$, **$P< 0.01$, ***$P< 0.001$, ****$P<0.0001$). WT, wildtype. N.D. not determined.

Supplementary Table S7. α-MSH-induced and PG-901-induced cAMP responses at WT and mutant MC5Rs and MC3Rs.

Mutation	α-MSH		PG-901		Cell surface expression (% WT)
	pEC_{50} ± S.E.M.	E_{max} ± S.E.M. (% WT MC5R)	pEC_{50} ± S.E.M.	E_{max} ± S.E.M. (% WT MC5R)	
**MC5R**					
WT MC5R	8.31 ± 0.02	100.00 ± 0.74	8.89 ± 0.04	100 ± 1.04	100.00
HA-MC5R(WT)-15AA-LgBiT	8.08 ± 0.11	92.99 ± 3.76	8.70 ± 0.10	103 ± 3.02	43.52 ± 11.62*
E92$^{2.60}$A	N.D.	N.D.	N.D.	N.D.	109.29 ± 6.55
E92$^{2.60}$Q	7.09 ± 0.17****	96.79 ± 3.86	7.4 ± 0.16****	87.25 ± 4.41	123.38 ± 14.01
I96$^{2.64}$A	N.D.	118.55 ± 2.6*	N.D.	113.49 ± 1.85*	78.17 ± 5.11
L99$^{2.67}$A	6.71 ± 0.09****	106.51 ± 4.79	7.88 ± 0.04****	105.54 ± 1.64	86.13 ± 5.13
N100^{ECL1}A	8.23 ± 0.09	105.62 ± 2.47	8.64 ± 0.09	98.44 ± 2.38	142.48 ± 35.87
D108$^{3.18}$A	8.13 ± 0.10	113.59 ± 3.19	8.70 ± 0.07	102.6 ± 2.07	98.9 ± 10.03
V111$^{3.21}$A	7.99 ± 0.10	109.92 ± 3.36	8.87 ± 0.10	103.02 ± 2.71	124.53 ± 7.39
D115$^{3.25}$A	5.88 ± 0.15****	104.94 ± 3.39	7.26 ± 0.10****	101.4 ± 1.78	70.71 ± 3.90
D115$^{3.25}$N	7.12 ± 0.09****	111.72 ± 1.15	7.93 ± 0.40***	98.15 ± 8.43	122.52 ± 11.59
N116$^{3.26}$A	7.91 ± 0.09*	104.62 ± 3.30	8.27 ± 0.09****	102.38 ± 2.98	106.14 ± 8.88
F118$^{3.28}$A	7.91 ± 0.09*	106.28 ± 2.74	8.30 ± 0.12****	103.65 ± 2.86	93.45 ± 5.76
D119$^{3.29}$A	5.66 ± 0.14****	124.38 ± 15.27***	6.40 ± 0.12****	93.5 ± 7.57	136.22 ± 19.07
D119$^{3.29}$N	5.58 ± 0.11****	119.59 ± 11.73*	5.81 ± 0.11****	73.72 ± 17.55***	84.79 ± 10.72
I122$^{3.32}$A	N.D.	97.19 ± 2.16	N.D.	89.24 ± 1.6	60.26 ± 15.97
V126$^{3.36}$A	7.51 ± 0.07****	102.09 ± 3.89	7.97 ± 0.09****	100.16 ± 4.9	86.65 ± 23.42
V126$^{3.36}$L	N.A.	N.A.	6.99 ± 0.26****	17.10 ± 3.65****	9.46 ± 0.89****
V126$^{3.36}$M	N.A.	N.A.	N.D.	N.D.	80.80 ± 2.36
I178$^{4.61}$A	6.20 ± 0.21****	88.08 ± 7.40	7.30 ± 0.14****	108.58 ± 4.47	79.99 ± 11.79
S181^{ECL2}A	7.81 ± 0.07**	104.30 ± 2.84	8.30 ± 0.12****	100.54 ± 3.79	95.80 ± 13.23
E182^{ECL2}A	8.11 ± 0.07	104.26 ± 2.28	8.4 ± 0.08**	102.17 ± 2.55	73.81 ± 6.91
V186$^{5.39}$A	7.95 ± 0.07*	101.62 ± 2.25	8.58 ± 0.10	100.2 ± 2.85	93.13 ± 11.61
I187$^{5.40}$A	7.59 ± 0.09****	103.49 ± 3.66	8.15 ± 0.12****	93.56 ± 3.87	71.59 ± 3.21
L190$^{5.43}$A	7.35 ± 0.16****	95.72 ± 4.87	7.68 ± 0.13****	107.84 ± 3.25	50.62 ± 13.82
F254$^{6.51}$A	6.10 ± 0.10****	114.22 ± 6.30	7.25 ± 0.10****	113.77 ± 4.53*	36.91 ± 11.35**
P265^{ECL3}A	8.06 ± 0.11	112.32 ± 3.27	8.69 ± 0.09	114.81 ± 2.86*	29.34 ± 9.00**
F277$^{7.35}$A	6.84 ± 0.11****	116.93 ± 5.62*	8.23 ± 0.07****	104.17 ± 2.37	104.60 ± 7.84
N278$^{7.36}$A	8.47 ± 0.06	120.78 ± 2.19**	8.96 ± 0.06	111.92 ± 1.89	30.80 ± 0.30**
L281$^{7.39}$A	6.35 ± 0.16****	93.23 ± 8.00	7.32 ± 0.06****	96.01 ± 2.91	116.01 ± 6.14
E92$^{2.60}$A/D115$^{3.25}$A/D119$^{3.29}$A	N.D.	N.D.	N.D.	N.D.	63.18 ± 4.38
**MC3R**					
WT MC3R	N.A.	N.A.	N.D.	N.D.	100.00
L128$^{3.36}$A	N.A.	N.A.	6.60 ± 0.08****	108.19 ± 5.01	69.28 ± 8.38

L128$^{3.36}$V	N.A.	N.A.	7.50 ± 0.11****	67.84 ± 3.50****	38.55 ± 15.54**
L128$^{3.36}$M	N.A.	N.A.	6.51 ± 0.55****	-5.76 ± 4.06****	81.22 ± 14.79

cAMP accumulation was normalized to the maximum response of wildtype (WT) MC5R and dose-response curves were analyzed using a three-parameter logistic equation to obtain pEC_{50} values. Cell surface expression was assessed by FACS and values were normalized to the WT (shown as percentage). The experiments were carried out independently at least three times. Data shown are means ± S.E.M. One-way ANOVA was used to determine statistical difference. N.D., not determined; N.A., not applicable. *$P < 0.05$, **$P < 0.01$, ***$P < 0.001$ and ****$P < 0.0001$.

Supplementary Table S8. Receptor binding profiles of α-MSH and PG-901 at MC5R.

Mutation	α-MSH		PG-901	
	pIC_{50} ± S.E.M.	Span ± S.E.M. (% WT)	pIC_{50} ± S.E.M.	Span ± S.E.M. (% WT)
WT	8.72 ± 0.07	100.00 ± 2.86	8.58 ± 0.06	100.00 ± 2.64
$I96^{2.64}A$	N.D.	N.D.	N.D.	N.D.
$L99^{2.67}A$	9.31 ± 0.37	18.31 ± 3.10****	9.13 ± 0.28	18.93 ± 2.40****
$D115^{3.25}A$	N.D.	N.D.	N.D.	N.D.
$F118^{3.28}A$	9.19 ± 0.45	24.20 ± 4.96****	8.66 ± 0.20	22.06 ± 1.93****
$D119^{3.29}A$	N.D.	N.D.	N.D.	N.D.
$I122^{3.32}A$	9.28 ± 0.10	93.50 ± 4.13	9.18 ± 0.15*	119.60 ± 8.32**
$V126^{3.36}A$	8.61 ± 0.21	43.91 ± 4.04****	8.28 ± 0.22	28.03 ± 2.79****
$I178^{4.61}A$	9.06 ± 0.34	14.15 ± 2.19****	N.D.	N.D.
$L190^{5.43}A$	8.68 ± 0.45	13.82 ± 2.75****	N.D.	N.D.
$F254^{6.51}A$	N.D.	N.D.	N.D.	N.D.
$F277^{7.35}A$	N.D.	N.D.	N.D.	N.D.
$L281^{7.39}A$	N.D.	N.D.	N.D.	N.D.
$E92^{2.60}A/D115^{3.25}A/D119^{3.29}A$	N.D.	N.D.	N.D.	N.D.

Whole cell binding assay was performed in HEK293 cells. Binding data were analyzed using a three-parameter logistic equation to determine pIC_{50} and span values. Data shown are means ± S.E.M. One-way ANOVA was used to determine statistical difference (*P< 0.05, **P< 0.01, ***P< 0.001, ****P<0.0001). WT, wildtype; N.D. not determined.

Reference

1. Ballesteros, J.A. & Weinstein, H. Integrated methods for the construction of three-dimensional models and computational probing of structure-function relations in G protein-coupled receptors. *Methods Neurosci.* **25**, 366-428 (1995).
